# The maternal KRAB-ZFP ZFPOBI1 reveals structural constraints governing ERV transcriptional co-option in mouse oocytes

**DOI:** 10.3389/fcell.2026.1851851

**Published:** 2026-07-14

**Authors:** Andrina J. Stäubli, Yan Huang, Louise J. H. Rasmussen, Freja Mosegaard Hansen, Hui Mun Loh, Michael J. Claxton, Daniel M. Messerschmidt

**Affiliations:** 1 Department of Cellular and Molecular Medicine, Faculty of Health and Medical Sciences, University of Copenhagen, Copenhagen, Denmark; 2 Institute of Molecular and Cell Biology (IMCB), Agency for Science, Technology and Research (A*STAR), Singapore, Singapore; 3 The Houston Methodist Research Institute, Department of Neurosurgery/Neuroprosthetics, Houston, TX, United States

**Keywords:** A430033K04Rik, endogenous retroviruses (ERVs), KRAB zinc finger proteins, LTR-initiated transcripts (LITs), oocyte-to-embryo transition, RLTR10, transcriptome, TRIM28 (KAP1)

## Abstract

Transposable elements (TEs) constitute a major fraction of mammalian genomes and play key roles in gene regulation, particularly during early development. Endogenous retroviruses (ERVs) are highly active in oocytes and early embryos, where their long terminal repeats (LTRs) can act as alternative promoters to generate LTR-initiated transcripts (LITs). Krüppel-associated box zinc finger proteins (KRAB-ZFPs) on the other hand repress TE activity in a sequence-specific manner through recruitment of the co-repressor TRIM28. Here, we identify the mouse KRAB-ZFP ZFPOBI1 as a previously uncharacterized, maternally expressed KRAB-ZFP that selectively targets the RLTR10 LTR subfamilies of the ERVK class. ZFPOBI1 binding is associated with robust TRIM28 recruitment and more modest changes in H3K9me3 enrichment at RLTR10 elements in mouse embryonic stem cells, consistent with canonical KRAB-ZFP-function. In oocytes, we show that RLTR10 elements contribute to LIT formation in a structure-dependent manner. While LTRs serve as transcriptional start sites, efficient splicing into downstream exons predominantly occurs via internal (-int) ERV sequences, indicating a functional separation of transcription initiation and RNA processing. Maternal deletion of *ZfpObi1* results in upregulation of a subset of RLTR10-driven LITs, demonstrating a role for ZFPOBI1 in restraining ERV-derived transcription. Notably, full-length RLTR10 elements are subject to additional KRAB-ZFP targeting at internal regions, suggesting that their repression is achieved through multilayered control. Consistent with this, the limited extent of transcriptional deregulation in *ZfpObi1*-deficient oocytes indicates partial functional redundancy within the KRAB-ZFP family. Together, our findings identify ZFPOBI1 as a regulator of RLTR10 elements and reveal how ERV structural organization constrains both transcriptional co-option and its epigenetic control in the oocyte transcriptome.

## 1 Introduction

Transposable elements (TEs) are repetitive sequences that comprise ∼40% of the mouse and up to ∼50% of the human genome (Consortium et al., 2002). Their expansion has profoundly shaped genome evolution, genome stability, and gene regulatory networks (Rebollo et al., 2010; Senft and Macfarlan, 2021). Endogenous retroviruses (ERVs) represent a subclass of TEs that have been extensively co-opted in host genomes as regulatory elements, including promoters, enhancers, long non-coding RNAs (lncRNAs), epigenetic scaffolds, and alternative exons. Through these functions, ERVs can remodel local transcriptional landscapes and give rise to chimeric transcripts (Friedman et al., 1996; Peaston et al., 2004; Friedli and Trono, 2015; Franke et al., 2017; Jachowicz et al., 2017; Brind’Amour et al., 2018). ERV activity has been demonstrated in embryonic stem cells (Macfarlan et al., 2012; Senft and Macfarlan, 2021) and is particularly prominent in oocytes and early embryos, where dynamic epigenetic reprogramming creates a permissive transcriptional environment also for ERVs (Peaston et al., 2004; Veselovska et al., 2015; Brind’Amour et al., 2018). In this context, ERV co-option contributes to developmental gene regulation and is required for proper progression from the gamete to the adult organism (Peaston et al., 2004; Franke et al., 2017; Sakashita et al., 2023).

Krüppel-associated box (KRAB) zinc finger proteins (ZFPs) have largely evolved as a host defence mechanism to domesticate and regulate endogenous retroviruses (ERVs) in mammalian genomes (Jacobs et al., 2014; Kauzlaric et al., 2017; Helleboid et al., 2019). With more than 400 members in mice, KRAB-ZFPs constitute one of the largest subfamilies of C2H2 zinc finger transcription factors (Urrutia, 2003; Kauzlaric et al., 2017). Their high abundance, combined with diverse spatial and temporal expression patterns, enables versatile and context-dependent repression of target sequences (Corsinotti et al., 2013; Kauzlaric et al., 2017). KRAB-ZFPs recognize target sequences in a sequence-specific manner through arrays of zinc finger domains (Emerson and Thomas, 2009; Isalan, 2013) while their N-terminal KRAB domain mediates recruitment of the co-repressor TRIM28 (also known as KAP1 or TIF1β) (Friedman et al., 1996). TRIM28 subsequently nucleates a repressive chromatin environment by recruiting the H3K9 histone methyltransferase SETDB1, heterochromatin protein 1 (HP1), the nucleosome remodelling and deacetylation (NuRD) complex, and DNA methyltransferases (DNMTs) (Schultz et al., 2001; 2002; Urrutia, 2003; Rowe et al., 2010; Quenneville et al., 2011).

During development, stage-specific modulation of ERV expression by KRAB-ZFP/TRIM28 complexes is essential. Maternal loss of *Trim28* results in severe developmental defects, including loss of imprinting, early male-specific lethality, and fully penetrant embryonic arrest (Messerschmidt et al., 2012; Lorthongpanich et al., 2013; Sampath Kumar et al., 2017). In contrast, individual KRAB-ZFPs studied to date are largely dispensable for development, likely reflecting extensive functional redundancy within this protein family (Wolf et al., 2015; Wolf et al., 2020; Ecco et al., 2016; Seah et al., 2019). Alternatively, the apparent dispensability may mask more subtle or context-dependent roles, including effects on transcriptional regulation, epigenetic inheritance, or genome stability that become apparent only under specific conditions or over longer developmental or generational timescales. Hence, and in the light of the severe maternal *Trim28* deletion phenotype, characterizing the pool of maternal KRAB zinc finger proteins and their targets is essential for understanding the maternal TRIM28/KRAB-ZFP network and its roles during the oocyte-to-embryo transition (OET), early embryogenesis, and epigenetic inheritance from germline to soma.

Here, we investigate a maternally highly expressed KRAB-ZFP encoded by *A430033K04Rik* (Jin et al., 2015). We show that ZFPOBI1 specifically targets a subset of RLTR10 family LTRs, where its binding is associated with recruitment of TRIM28 and enrichment of H3K9me3. We further examine the contribution of RLTR10 elements to LTR-initiated transcripts (LITs) in oocytes and assess the LIT regulation in the presence or absence of maternal *ZfpObi1*. Indeed, loss of *ZfpObi1* results in transcriptional deregulation of RLTR10-driven LITs, highlighting its role in restraining ERV-derived transcription.

## 2 Materials and methods

### 2.1 Mice

*ZfpObi1* knockout mice (*C57BL/6J-Obi1* ^*em1Meschmi*^ */J)* were established by CRISPR-Cas9 with the Core Facility for Transgenic Mice at the University of Copenhagen using gRNAs targeting the 5′ and 3′ ends of exon 4 (IDT, Alt-R CRISPR-CAS9 sgRNA, Supplementary Table S18). Founder mice were identified by genotyping (for primer sequences see Supplementary Table S18). After confirmation of the deletion by Sanger sequencing mice were backcrossed with a wild-type C57BL/J mouse. Mice used in experiments were re-genotyped to confirm identity. All mouse work was approved by the Danish Animal Ethical Committee (“Dyreforsøgstilsynet”) and is performed according to the national ethical animal research regulations.

### 2.2 Oocyte and ovary isolation

For RNA-sequencing GV oocytes were harvested from ovary follicles by scratching ovaries from euthanized females in M2 medium (Sigma, M7167) with a needle. Fully grown (∼70 µm) oocytes with clear germinal vesicles were washed in droplets of M2 to remove attached cumulus cells (Behringer, 2014). Mouse ovary RNA was extracted from wildtype C57BL/J mice using Trizol (Invitrogen, 15596062) and chloroform. RNA was collected from the aqueous phase after centrifugation in phasemaker tubes (Invitrogen A33248) and purified using RNeasy Mini Kit (Qiagen, 74106). 50 µM Oligo D(T)12-18 Primers (Invitrogen, 18418012) were annealed to RNA at 65 °C for 5 min and reverse transcribed into cDNA using SuperScriptIV (Invitrogen, 18090010) at 50 °C for 10 min before inactivation at 80 °C for 10 min.

### 2.3 *ZfpObi1* and luciferase reporter cloning

Full-length *ZfpObi1* (*A430033K04Rik,* isoform: ENSMUST00000069862.10) and *ZfpObi1-ΔKRAB* were cloned from mouse ovary cDNA into PJ549 piggy-bac vector (DNA2.0) previously modified with chicken β-actin promoter and N-terminal triple HA tag using *Not*I (NEB, R0189S) restriction sites (Seah et al., 2019). For luciferase reporter assays respective RLTR10 elements were PCR amplified from mESC gDNA and inserted into a pGL4.23 vector (Promega E8411) modified with a PGK promoter to drive luciferase activity using *Kpn*I (NEB, R3142S) and *Xho*I (NEB, R0146S) (Seah et al., 2019). For primer sequences see Supplementary Table S18.

### 2.4 Tissue culture and transfection

E14 mESC were cultured in 2i+LIF serum-free medium under standard conditions on 0.1% gelatine coated dishes. Cells were passaged every 2-3 days. For luciferase assays HEK293T cells were cultured in DMEM (Gibco, 41966-029), 10% FBS (Gibco, 26140079), and 1% Pen/Strep (Gibco, 15140). To establish stable cell lines E14 or HEK293T cells were transfected with respective constructs using Lipofectamine 3000 (Invitrogen, L3000001) and were selected with 1 μg/mL puromycin (Gibco, A1113803).

### 2.5 Co-immunoprecipitation and Western blotting

Cells were lysed in RIPA buffer (150 mM NaCl, 50 mM Tris-HCl pH8, 1 mM EDTA, 1% Triton X-100, 0.1% sodium deoxycholate, supplemented with cOmplete Mini EDTA-free protease inhibitor (Roche, 11836170001)). Protein concentration was assessed by Bradford Protein Assay (Bio-Rad, 5000205). For IP 500 µg protein lysate was incubated with 1 µg of mouse anti-HA (BioLegened, 901533, 1:2,000) antibodies at 4 °C overnight. 50 µL pre-blocked Recombinant Protein G-Sepharose 4B beads (ThermoFisher Scientific, 101242) were added and incubated for 3 h. Samples were washed three times in RIPA buffer and immunoprecipitated material was eluted with 2x Laemmli buffer (Bio-Rad, 1610737EDU) and denatured for 5 min. Eluted samples or 50 µg protein lysates for Western blot were run on a 10% SDS-PAGE gel and transferred to a nitrocellulose membrane. Membranes were blocked, incubated with primary antibodies [mouse α-HA (BioLegened, 901533, 1:2,000), mouse α-TRIM28 (Abcam, ab22553, 1:5,000), or mouse α-β-actin (Abcam, ab8226, 1:5,000)] overnight at 4 °C and secondary antibodies HRP-conjugated anti-rabbit (Cytiva NA934) or HRP-conjugated anti-mouse (Cytiva NA931) at 1:10,000 for 1 h at room temperature. Proteins were visualized using Immobilon Western Chemiluminescent HRP Substrate (Merck, WBKL S0500).

### 2.6 Luciferase reporter assay

Luciferase reporter assays were performed in stably transfected *3xHA-ZfpObi1*, *3xHA-ZfpObi1-ΔKRAB, 3xHA-vector* HEK293T cells. In 96-well plates, cells were transfected with RLTR10-pGL4.23 (30 ng) constructs and Renilla control vector pGL4.75 (20 ng) (Promega E6931) using Lipofectamin3000 (Invitrogen, L3000001). 24 h after transfection cells were lysed and luciferase activity was measured using the Dual-Glo Luciferase Assay System (Promega, E2920) according to the manufacturer’s protocol. Experiments were performed in triplicates and repeated at least 4 times for each construct. Luciferase expression was normalized to renilla measurement and luciferase activity relative to *3xHA-vector* control was calculated. Statistical analysis using ordinary one-way ANOVA was done in *Prism10* (GraphPad Software, CA), p-value <0.05 was considered significant.

### 2.7 Chromatin immunoprecipitation and sequencing (ChIP-seq)

ChIP-seq was performed on three biological replicates of *3xHA-ZfpObi1*, and *3xHA-vector* control expressing E14 mESCs as previously described in Han et al. (2020). In brief, cells were crosslinked using 1% formaldehyde for 10 min at room temperature, quenched with 125 mM glycine and washed in cold PBS. Chromatin extraction from nuclei was performed using LB1 buffer (50 mM HEPES-KOH pH7.5, 140 mM NaCl, 1 mM EDTA, 10% glycerol, 0.5% NP-40, and 0.25% Triton X-100), LB2 (LB2 (10 mM Tris-HCl pH8.0, 200 mM NaCl, 1 mM EDTA, 0.5 mM EGTA), and LB3 (10 mM Tris-HCl pH8.0, 100 mM NaCl, 1 mM EDTA, 0.5 mM EGTA, 0.1% sodium deoxycholate, 0.5% N-lauroylsarcosine), supplemented with 0.2 mM PMSF and protease inhibitor cocktail (Roche, 11836170001). Chromatin DNA was sonicated to 100–500 bp using the Branson Digital Sonifier (Branson Ultrasonics, Danbury, Connecticut) at 30% amplitude for four cycles. Sonicated chromatin was cleared by centrifugation, and immunoprecipitations were performed using 50 µg chromatin and 5 µg rabbit α-HA (Abcam, ab9110), rabbit α-TRIM28 (Abcam, ab10484), or rabbit α-H3K9me3 (Abcam, ab8898) overnight at 4 °C. 50µL of pre-blocked Recombinant Protein G–Sepharose 4B beads (ThermoFisher Scientific, 101242) in LB3 was and incubated for 4 h at 4 °C. Beads were washed in low salt buffer (0.1% SDS, 1% Triton X-100, 2 mM EDTA and 20 mM Tris-HCl pH8), high salt buffer (0.1% SDS, 1% Triton X-100, 2 mM EDTA, 20 mM Tris-HCl pH8 and 500 mM NaCl), and LiCl buffer (0.25M LiCl, 1% NP-40, 1% sodium deoxycholate, 1 mM EDTA and 10 mM Tris-HCl pH8). DNA was eluted (50 mM Tris-HCl pH8.0, 10 mM EDTA, 1% SDS) for 15 min at 30 °C and reverse crosslinked over night with 0.5 mg/mL Rnase A (Sigma, RNASEA-RO) overnight at 65 °C. The DNA was purified using the QIAquick PCR purification kit (Qiagen 28104) and concentrations were measured using Qubit dsDNA high sensitivity Assay (Invitrogen Q32851). ChIP-seq libraries were made with the NEBNext Ultra II DNA Library Preparation Kit for Illumina (NEB E7645S) following manufacturer’s instructions. PCR amplification was performed for 12 cycles with 1 ng input DNA. Libraries were quantified using Qubit and Agilent High Sensitivity DNA kit (Agilent, 5067-4626). Final 4 nM library pools were sequenced on an Illumina NextSeq2000 using single-end 100 bp reads to >15 million reads per library. Two libraries below 5 million reads were excluded from the analysis (01_3xHA-ZfpObi1_HA, 01_3xHA-vec_H3K9me3).

### 2.8 ChIP-seq analysis

Generated ChIP-seq libraries were processed using *nf-core* v.2.13.1a (https://nf-co.re/) *ChIP-seq pipeline* v.2.0.0 based on *Nextflow* v.23.10.1 --aligner bowtie2 --readlength 100 --genome mm10 (RefSeq annotation; from iGenomes, https://support.illumina.com/sequencing/sequencing_software/igenome.html). 3HA-ZFPOBI1 peaks were called using MACS2 peak calling--narrow_peak true over input samples (Zhang et al., 2008; Tommaso et al., 2017; Ewels et al., 2020). High confidence peaks for HA ChIP-seq were generated using two remaining replicates and *bedtools Intersect intervals* Galaxy-v2.30.0 -wa on the *usegalaxy.eu* public platform (Quinlan and Hall, 2010; Galaxy Community, 2024). Identification of HCPs overlapping repetitive elements (RE) (RepeatMasker, Smit AFA, Hubley R, Green P*, RepeatMasker* Open-3.0 http://www.repeatmasker.org. 1996-2010, downloaded from UCSC Genome Browser) (Jurka et al., 2005) was done using *bedtools Intersect intervals* Galaxy-v2.30.0 -a HCP summits ±35 bp -b mm10.repeats.bed -wao -f 0.25 -F 0.9. Motif analysis was performed on peak summits ±15 bp of respective “overlap categories” HCPs using STREME on *MEME Suite* v5.5.7 (https://meme-suite.org/meme/index.html) (Machanick and Bailey, 2011; Bailey, 2021). Motif predictions were done using https://zf.princeton.edu/index.php (Persikov and Singh, 2014). Binding motif occurrences were identified using *FIMO* on *MEME Suite* v5.5.7 (https://meme-suite.org/meme/index.html) (Grant et al., 2011; Bailey et al., 2015), Enrichment profiles were assessed and visualized using *EaSeq* v1.20 (http://easeq.net) (Lerdrup et al., 2016). Individual replicates were pooled for visualization. ChIP-seq datasets are available under GSE326899.

### 2.9 Curation of RLTR10 ERV elements and classification of solo versus full-length insertions

Repeat annotations for the mouse genome (mm10) were obtained from RepeatMasker (Jurka et al., 2005). To reduce fragmentation inherent to RepeatMasker annotations, adjacent repeat fragments were merged in a strand-specific manner using BEDTools implemented in Galaxy. Relevant fragmented ERV annotations (RLTR10, RLTR10A, RLTR10D) were stitched using mergeBed with parameters -s -d 50, merging features on the same strand separated by ≤50 bp. Respective internal (“-int”) elements (RLTR10-int, IAP-d-int) were merged analogously using a more permissive distance threshold (-s -d 100) to account for increased fragmentation of internal regions. Where required, original annotation attributes were retained using -c/-o collapse. Merging was performed independently for each subfamily to preserve annotation specificity, resulting in a curated, strand-resolved set of LTR and internal elements. Proximity of LTRs to internal (-int) regions was determined by BEDTools (closestBED) implemented on galaxy. To classify RLTR10 elements into solo LTRs, truncated insertions, and full-length ERVs, curated LTR and internal annotations were combined and analyzed at the locus level. LTR and internal datasets were concatenated, annotated with feature type (LTR or INT), and encoded with binary indicators (isLTR, isINT). The combined dataset was sorted and clustered using BEDTools *cluster* with strand specificity and a maximum distance of 50 bp (-s -d 50), grouping nearby features into putative ERV loci. Clusters were summarized by chromosome, strand, family key, and cluster ID, collapsing genomic coordinates to the minimum start and maximum end, and summing feature indicators. Loci were classified based on their composition: clusters containing 5′ and 3′ LTR elements and one or more internal element (-int) fragments were defined as full-length ERVs (LTR–int–LTR configuration); clusters containing a single LTR and ≥1 internal element were classified as truncated insertions; clusters containing LTR elements without respective internal sequence were classified as solo LTRs; clusters containing only internal sequence were annotated as internal-only fragments. Final locus coordinates were reported as strand-specific BED intervals.

All analyses, unless otherwise indicated, were performed using Galaxy workflows, employing standard tools (Cut columns, SortBED, mergeBed, cluster, Concatenate datasets, and Group-by aggregation). Where required, composite keys were generated to enable multi-field grouping, and numerical formatting artifacts (e.g., scientific notation) were corrected prior to downstream analysis.

### 2.10 RNA-sequencing and analysis

Single oocyte RNA-sequencing was performed as previously described (Han et al., 2020) following the smart-seq2 protocol (Picelli et al., 2014). Minimal adjustments to the protocol were made: GV oocytes were PCR preamplified for 10 cycles. The tagmentation reaction was performed on 1 ng of cDNA per reaction using the Illumina Nextera XT DNA sample preparation kit (Illumina, FC-131-1096). NGS libraries were amplified for 10 cycles, pooled and sequenced Illumina NextSeq2000 using 100 bp single-end reads to >15 million reads per sample. RNA-seq libraries were mapped using the nf-core v2.13.1a (https://nf-co.re/) RNA-seq pipeline v3.14.0 based on Nextflow v23.10.1 (Tommaso et al., 2017; Ewels et al., 2020) using: --aligner star_salmon to--genome mm10/GRCm38.

For GV Oocyte-specific transcriptome assembly RNA-seq data from oocytes (public dataset wildtype GV oocytes (Seah et al., 2025) or *ZfpObi1* KO/control GV oocytes, respectively) were processed using a StringTie-based transcriptome assembly and quantification workflow. Per-sample transcriptomes were assembled using StringTie with guidance from the reference annotation. Individual assemblies were subsequently merged into a unified transcriptome using the *StringTie-merge* function, generating a comprehensive annotation across all samples. The merged transcriptome was compared to the reference annotation using *gffcompare* to classify transcript features.

LTR-initiated transcripts (LITs) were identified using a custom pipeline integrating transcript structure and repeat annotations. Transcript models derived from the merged and annotated transcriptome (gffcompare output) were parsed to extract the first exon, transcription start site (TSS; 1 bp, strand-aware), and the last nucleotide of the first exon using custom Python scripts. Resulting genomic features were converted to BED format and intersected with RepeatMasker-based repeat annotations (mm10) using *BEDtools* (Quinlan and Hall, 2010) to determine overlap of TSSs and first-exon termini with repetitive elements. Transcripts were classified as LITs if their TSS overlapped an LTR/ERV element. To further characterize transcript structure, overlap of the first exon terminus with repeat annotations was used to distinguish cases where transcription initiates within an LTR but splicing occurs from other downstream regions (e.g., ERV internal regions). These annotations were integrated into a unified table describing repeat-associated first-exon architecture. Finally, single-exon transcripts were flagged to distinguish potential artefacts or fully repeat-derived transcripts. Redundant transcript isoforms were collapsed based on shared, identical first-exon structure to generate a non-redundant set of LIT loci. The final output consisted of a curated table of LITs with associated repeat annotations and exon structure information, used for downstream analyses.

Differential expression analysis was performed within the Galaxy platform (Galaxy Community, 2024). Aligned RNA-seq reads (coordinate-sorted BAM files generated with STAR) were quantified using *featureCounts* (Liao et al., 2014) (Galaxy Version 2.0.3) against the custom merged transcriptome annotation generated by StringTie (see above). Read counting was performed using default parameters, retaining uniquely mapping reads and without strand specificity, consistent with the library preparation protocol. Gene-level count matrices were used as input for differential expression analysis with *DeSeq2* (Love et al., 2014) (Galaxy Version 2.11.40.8), using default settings. Counts were normalized using the median-of-ratios method, and differential expression between wild-type (WT) and *ZfpObi1* knockout (KO) samples was assessed using a negative binomial generalized linear model. Statistical significance was determined using adjusted p-values (FDR, Benjamini–Hochberg correction). Genes were considered significantly differentially expressed at FDR ≤ 0.05, and a minimum expression threshold (baseMean ≥20) was applied to exclude low-abundance genes. Plots and heatmaps were generated using GraphPad Prism version 10 (GraphPad Software, CA). Aligned reads with splice junctions were visualized on the IGV browser (Robinson et al., 2011). BigWig coverage files were generated using *deeptools bamCoverage* v3.5.5 and--binSize 50 --normalizeUsing RPKM (Quinlan and Hall, 2010). RNA-seq datasets are available under GSE326965.

### 2.11 Data use and availability

RNA-seq data sets generated in this study have been deposited in NCBI’s Gene Expression Omnibus (GEO) under accession code GSE326965. Source data are shared via GEO (GSE326965) or are provided within this paper in supplementary tables. ChIP-seq data sets generated in this study have been deposited in GEO under accession code GSE326899. Source data are shared via GEO (GSE326899) or are provided within this paper in supplementary tables. Published GV oocyte RNA-seq datasets are available in GEO, GSE126688 (Seah et al., 2019) or GSE283055 (Seah et al., 2025) and were re-analysed using the described pipelines above. Ribo-lite and mRNA-seq FPKM values of all embryonic stages were taken from GSE165783 (Xiong et al., 2022). Raw ZFP932 and GM15446 ChIP-seq data were obtained from GSE74278 and reprocessed as described above for enrichment analysis.

## 3 Results

### 3.1 ZFPOBI1 is a bona fide, maternal KRAB-ZFP

**FIGURE 1:**
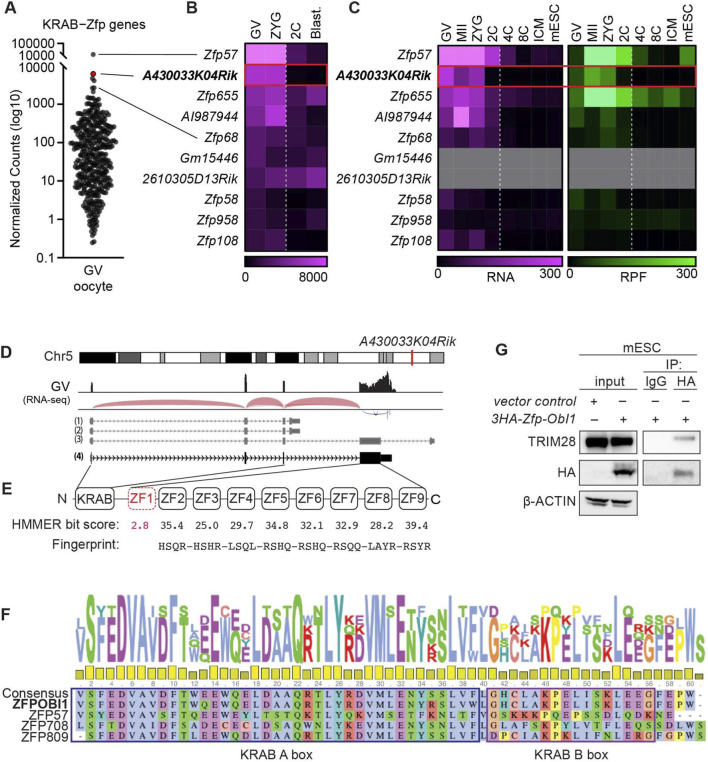
*ZFPOBI1* is a maternal KRAB-zinc finger protein. **(A)** Beeswarm plot showing normalized expression counts of annotated and predicted murine KRAB-ZFP genes in wild-type GV oocytes (GSE126687) (Seah et al., 2019). *A430033K04Rik* is highlighted in red. **(B)** Heatmap of normalized expression counts for the ten highest expressed KRAB-ZFP genes across germinal vesicle oocytes (GV), zygotes (ZYG), 2-cell (2C), and blastocyst (Blast.) stages (GSE126687) (Seah et al., 2019). *A430033K04Rik* is highlighted (red box). **(C)** Heatmaps showing RNA expression (left; FPKM) and ribosome-protected fragments (RPF; right) for the same top ten KRAB-ZFP genes across oocyte and preimplantation stages (GV, MII, ZYG, 2C, 4C, 8C) (GSE165782) (Xiong et al., 2022). *A430033K04Rik* is highlighted (red box). **(D)** Genome browser view of RNA-seq coverage in wild-type GV oocytes at the *A430033K04Rik* locus, including four annotated GENCODE transcript isoforms (ENSMUST00000032590.13 (1), ENSMUST00000200521.4 (2), ENSMUST00000198958.4 (3), ENSMUST00000069862.10 (4)). **(E)** Schematic representation of the ZFPOBI1 protein encoded by ENSMUST00000069862.10. The N-terminal KRAB domain and nine C2H2 zinc fingers (ZFs) are shown. HMMER bit scores ≥20 indicate canonical zinc fingers, whereas lower scores denote degenerate domains (ZF1, highlighted in red). The predicted DNA-contacting residues (positions −1, 2, 3, and 6) are indicated (“fingerprint”). **(F)** Multiple sequence alignment of KRAB domain amino acid sequences from ZFPOBI1 and known TRIM28-interacting KRAB-ZFPs (ZFP57, ZFP708, ZFP809), showing conservation of KRAB-A and KRAB-B domains. **(G)** Co-immunoprecipitation of ZFPOBI1 and TRIM28 in mESCs. Input blots confirm expression of 3xHA-ZFPOBI1 and TRIM28 in control and ZFPOBI1-expressing cells. Immunoprecipitation with anti-HA antibody (or IgG control) followed by Western blotting demonstrates interaction between ZFPOBI1 and TRIM28.

Krüppel-associated box (KRAB) zinc finger proteins (KRAB-ZFPs) display diverse tissue and developmental expression patterns. To identify candidate factors involved in targeting TRIM28 complexes to maternal and early embryonic epigenetically sensitive regions, we analysed KRAB-ZFP expression in GV oocytes (Seah et al., 2019) (Figure 1). We generated a manually curated list of 486 putative murine KRAB-ZFPs genes, including canonical KRAB-ZFPs and KRAB-ZFP-like proteins identified based on the presence of zinc finger domains and proximity to KRAB domains (Supplementary Table S1) (Kauzlaric et al., 2017). As expected, *Zfp57*, a key regulator of imprint maintenance during early development, was the most highly expressed KRAB-ZFP in oocytes (Li et al., 2008; Mackay et al., 2008; Quenneville et al., 2011). Notably, the second most highly expressed KRAB-ZFP gene in GV oocytes was *A430033K04Rik* (Figure 1A). Expression profiling across the oocyte-to-embryo transition (OET) and early embryonic stages revealed that *A430033K04Rik* is highly expressed in oocytes but sharply downregulated following embryonic genome activation (EGA) at the two cell stage (Figure 1B) (Seah et al., 2019). This expression pattern is shared with other top candidates such as *Zfp58* and *Zfp108*, and contrasts with KRAB-ZFPs that remain expressed after EGA, including *Zfp57*, *Zfp655*, and *Gm15446* (Figure 1B). The maternal specificity of *A430033K04Rik* expression is further supported by independent RNA-seq and ribosome profiling data, which show robust expression and translation in oocytes and zygotes but not at later stages nor in mouse embryonic stem cells (Figure 1C) (Xiong et al., 2022).

*A430033K04Rik* was initially identified as a protein-coding gene in mouse ovaries in the RIKEN project (Hayashizaki, 2003) and subsequently characterized as a nuclear KRAB-ZFP with repressive activity (Jin et al., 2015). More recently, it has also been implicated in osteoblast differentiation *in vitro* and named Osteoblast Inducer 1 (ObI1) accordingly (Querques et al., 2019). We will henceforth refer to *A430033K04Rik* as *ZfpObi1*. Unlike many KRAB-ZFP genes, *ZfpObi1* is not located within a KRAB-ZFP gene cluster but resides in relative isolation on chromosome 5, adjacent to *Zfp68*, another maternally expressed KRAB-ZFP (Kauzlaric et al., 2017; Wolf et al., 2020) (Figure 1A). However, *Zfp68* and *ZfpObi1* are not paralogs (based on sequence and “finger print” conservation), and no additional paralogs of *ZfpObi1* were identified genome-wide. Four transcript isoforms are annotated in GENCODE (Frankish et al., 2021) of which two (ENSMUST00000198958.4 and ENSMUST00000069862.10) are predicted to encode canonical C2H2 zinc finger proteins (Figure 1D). Of these, only the RefSeq-validated transcript (NM_183025; ENSMUST00000069862.10) was detected in oocyte RNA-seq data (Figure 1D) (Seah et al., 2019). This transcript encodes a 680 amino acid KRAB-ZFP protein containing an N-terminal KRAB domain (encoded by exons 1–3), comprising KRAB-A and KRAB-B subdomains, and a C-terminal array of nine zinc fingers encoded by exon 4 (Figure 1E). The first zinc finger (ZF1) appears degenerate, lacking the canonical zinc-coordinating cysteines and exhibiting a low HMMER bit score (<20), whereas ZFs 2–9 are well conserved and display sequence features consistent with functional DNA-binding domains (Isalan, 2013; Persikov and Singh, 2014) (Figure 1E). The KRAB domain, which mediates TRIM28 interaction (Urrutia, 2003), is highly conserved relative to other TRIM28-interacting KRAB-ZFPs (Figure 1F) (Li et al., 2008; Wolf and Goff, 2009; Jin et al., 2015; Seah et al., 2019). Consistent with this, co-immunoprecipitation of 3xHA-tagged ZFPOBI1 confirms robust interaction with TRIM28 (Figure 1G).

These results establish ZFPOBI1 as a *bona fide*, maternally expressed KRAB-ZFP that interacts with TRIM28 and is predicted to function as a transcriptional repressor in oocytes.

### 3.2 ZFPOBI1 target discovery

**FIGURE 2:**
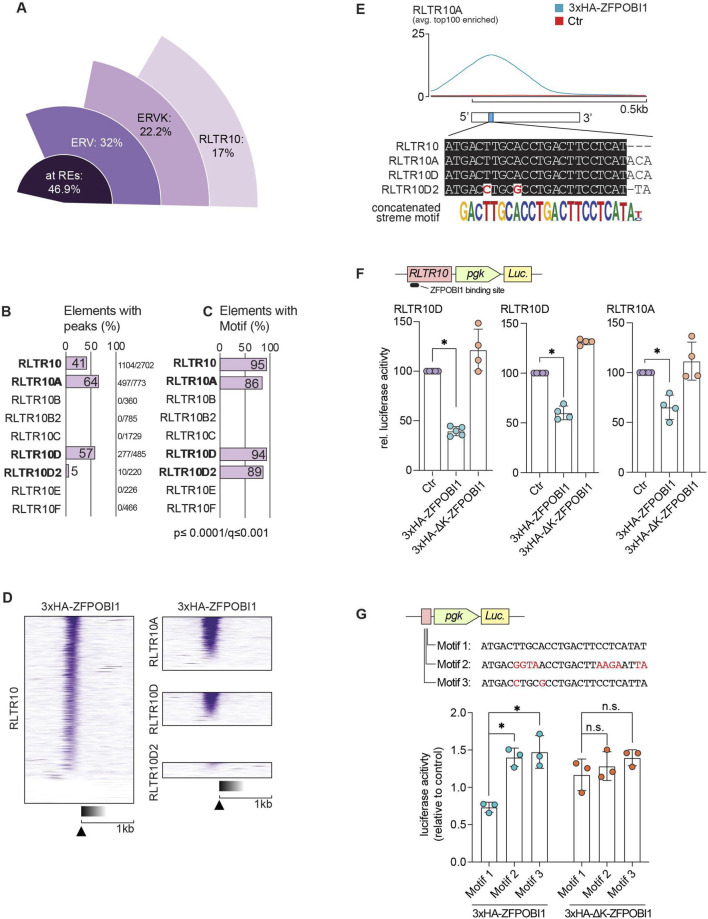
ZFPOBI1 selectively binds RLTR10 LTR subfamilies and represses transcription through a sequence-specific motif. **(A)** Hierarchical breakdown of HCPs overlapping repetitive elements, highlighting enrichment at ERVK elements and a prominent contribution of RLTR10 subfamilies. **(B)** Fraction of ZFPOBI1 HCPs overlapping RLTR10 subfamilies, shown as percentage of total elements per subfamily (counts indicated). **(C)** Fraction of elements containing the ZFPOBI1 motif, as identified by FIMO. Match significance was determined using the FIMO P-value (≤0.0001) and Benjamini–Hochberg corrected q-value (≤0.001). Binding and motif occurrence are enriched in RLTR10 subfamilies classified as “group a” (in bold), but largely absent from “group b” elements. **(D)** Heatmaps showing ZFPOBI1 ChIP–seq signal across curated RLTR10 “group a” elements and individual subfamilies, aligned at the 5′ LTR boundary (±1 kb). Strong enrichment is observed across RLTR10, RLTR10A and RLTR10D elements, but is reduced or absent at RLTR10D2. **(E)** ZFPOBI1 ChIP–seq metaprofile across RLTR10A elements (top 100 enriched), aligned at the 5′ LTR boundary, with control. Sequence alignment reveals a conserved motif in RLTR10, RLTR10A and RLTR10D, with divergence in RLTR10D2; the concatenated ZFPObI1 binding motif is shown below. **(F)** Luciferase reporter assays using RLTR10-derived sequences in HEK293T cells expressing control, 3xHA-ZFPOBI1, or a KRAB domain deletion mutant (3xHA-ΔKRAB-ZFPOBI1). **(G)** Luciferase reporter assays using the consensus RLTR10 ZFPOBI1 binding motif (top), a mutated motif (middle), or the RLTR10D2 motif variant (bottom). ZFPOBI1-dependent repression requires both an intact binding motif and the KRAB domain, whereas motif disruption or the two RLTR10D2-specific sequence variants abolish repression. **(F, G)** Data represent mean ± SD from four and three independent experiments, respectively. Statistical significance was assessed by one-way ANOVA with multiple comparisons; *p ≤ 0.05 was considered significant.

To identify genomic binding sites of ZFPOBI1, we performed chromatin immunoprecipitation followed by sequencing (ChIP-seq) in mouse embryonic stem cells (mESCs) overexpressing *3xHA-ZfpObi1*. Owing to their experimentally tractable nature and accessible chromatin landscape, heterologous expression of KRAB-ZFPs in mESCs or similar provide a useful platform for defining their binding specificity and associated TRIM28 recruitment (Wolf and Goff, 2009; Imbeault et al., 2017; Seah et al., 2019; Wolf et al., 2020). Our analysis identified >11,000 high-confidence peaks (HCPs), defined as the intersection of two independent experiments (Supplementary Table S2). These peaks were highly specific to 3xHA-ZFPOBI1, as confirmed by comparison to a published ChIP-seq dataset for 3xHA-ZFP708, which targets RMER19B ERVs (Seah et al., 2019) (Supplementary Figure S1A). Given the well-established role of KRAB-ZFP/TRIM28 complexes in targeting transposable elements (Wolf and Goff, 2009; Rowe et al., 2010; Ecco et al., 2016; Seah et al., 2019; Wolf et al., 2020) we intersected ZFPOBI1 HCPs with annotated repetitive elements. Approximately 50% of HCPs overlapped repetitive elements (REs), of which ∼90% corresponded to transposable elements (TEs) (Supplementary Figure S1B; Supplementary Table S2). The largest fraction of TEs underlying peaks belongs to the RLTR10 family, which represents a large, ERV LTR retrotransposon family in the mouse genome (Figure 2A; Supplementary Figure S1B) (Kawase and Ichiyanagi, 2023).

The large number of peaks and their distribution across multiple genomic categories prompted a detailed motif analysis across the six most prominent peak-associated categories (non-repetitive regions, simple/low-complexity repeats, LINEs, SINEs, MaLRs, and RLTR10-related LTRs, here referred to as RLTR10*-LTRs). Motif discovery using STREME (Machanick and Bailey, 2011; Bailey, 2021) (Supplementary Figure S1C) identified a minimal “core” motif (CCTC) that was centrally enriched across peaks in all categories, but most prominently within RLTR10*-LTR-associated HCPs (92% of peaks) (Supplementary Figure S1C; Supplementary Table S2). In addition, a second, highly conserved motif was identified in 95% of RLTR10*-LTR-overlapping HCPs (Supplementary Table S2). Using “FIMO” (Find Individual Motif Occurrences) (Grant et al., 2011) this motif was found immediately upstream of the CCTC core motif and was largely absent from peaks in the other genomic categories (Supplementary Figure S1E; Supplementary Table S4). Concatenation of the two motifs, based on enrichment and positional information, yielded a composite binding sequence closely matching the *in silico* predicted ZFPOBI1 binding motif (Persikov and Singh, 2014) (Supplementary Figure S1F). Accordingly, one may suggest ZFPOBI1 has evolved to specifically recognize RLTR10*-LTR sequences. At the same time, the widespread presence of the minimal CCTC core motif across genomic contexts suggests that partial engagement of the zinc finger array, potentially involving ZFs 6 and 7, may mediate lower-affinity binding outside RLTR10 elements in our ectopic overexpression system in mESCs (Supplementary Figure S1F).

### 3.3 ZFPOBI1 specifically targets RLTR10-LTR sub-families

RLTR10*-LTRs represent the most abundant repeat class underlying ZFPOBI1 high-confidence peaks (HCPs) (Figure 2A). These elements belong to the ERVK family of retrotransposons and are closely related to intracisternal A-type particles (IAPs) and MMERVK elements, which play prominent roles in mouse genome regulation (Kawase and Ichiyanagi, 2023). RLTR10-type LTRs can be divided into two evolutionary branches. RLTR10-, RLTR10A-, RLTR10D-, and RLTR10D2-LTRs (hereafter referred to as “group a”) are related to MMERVK10D3_LTR elements, whereas RLTR10B-, RLTR10B2-, RLTR10C-, RLTR10E-, and RLTR10F-LTRs (“group b”) are related to MMERVK10C_LTR elements (Kawase and Ichiyanagi, 2023). To investigate the relationship between ZFPOBI1 and RLTR10 LTRs in detail, we curated Repbase annotations (Jurka et al., 2005) by merging adjacent fragments of the same subfamily (≤50 bp apart) in the same orientation, thereby approximating individual LTR loci derived from fragmented repeat annotations (Supplementary Tables S4, S5). We then assessed the distribution of ZFPOBI1 HCPs across all curated RLTR10*-LTRs.

Strikingly, ZFPOBI1 binding was restricted to “group a” subfamilies, with peaks observed at RLTR10- (41% of elements bound), RLTR10A- (64%), and RLTR10D-LTRs (57%). In contrast, no peaks were called at RLTR10B-, RLTR10B2-, RLTR10C-, RLTR10E-, or RLTR10F-LTRs (0% bound) (Figure 2B). Motif analysis using FIMO with the composite RLTR10 motif (Supplementary Figure S1F) further supported this specificity, revealing strong enrichment of motif occurrences within the same RLTR10 subfamilies (Figure 2C; Supplementary Table S7).

This subfamily-specific targeting is also reflected in ChIP-seq signal profiles, with strong 3xHA-ZFPOBI1 enrichment across “group a” elements, but little to no enrichment at “group b” LTRs (Figure 2D; Supplementary Figure S1G, respectively). A notable exception is RLTR10D2-LTRs, which cluster within “group a” but exhibit minimal ZFPOBI1 binding (5% of elements bound) (Figure 2B) and only weak enrichment (Figure 2D), despite a high frequency of predicted motif occurrences (89% of 220 curated elements) (Figure 2C; Supplementary Table S7).

Alignment of RLTR10*-LTR consensus sequences revealed strong conservation within each group and substantial divergence between groups, consistent with their distinct evolutionary origins (Kawase and Ichiyanagi, 2023) (Supplementary Figure S2). This divergence is mirrored by the presence or absence of the ZFPOBI1 binding motif, which is conserved in “group a” but absent in “group b” elements (Supplementary Figure S2). Notably, the predicted ZFPOBI1 binding motif is conserved across RLTR10, RLTR10A, and RLTR10D consensus sequences and is consistently located in the 5′ region of the LTR, where ChIP-seq signal is enriched. In contrast, sequence divergence at this site in RLTR10D2 elements likely underlies their reduced ZFPOBI1 binding (Figure 2E).

These results demonstrate that ZFPOBI1 selectively targets a defined subset of RLTR10 LTRs, with binding specificity shaped by subfamily-specific sequence features.

### 3.4 ZFPOBI1 is a transcriptional repressor for specific RLTR10 LTRs

Having defined ZFPOBI1 target sites, we next assessed the transcriptional consequences of its binding. To this end we performed reporter assays in HEK293T cells as a heterologous reporter system, allowing assessment of the intrinsic repressive capacity of ZFPOBI1 independent of endogenous murine regulatory networks. To test whether ZFPOBI1 imposes a repressive state at RLTR10 LTRs, we cloned three representative ZFPOBI1-bound LTRs (two RLTR10D and one RLTR10A) into luciferase reporter constructs (Seah et al., 2019). Luciferase activity was tested in cells co-expressing either 3xHA-ZFPOBI1, a KRAB-domain deletion mutant (3xHA-ΔK-ZFPOBI1) or an “empty” control vector. Expression of ZFPOBI1 resulted in a significant reduction in luciferase activity for all three LTR constructs compared to control conditions (Figure 2F). In contrast, expression of the KRAB-deleted variant failed to repress reporter activity, with luciferase levels comparable to controls.

To determine whether repression depends on direct recognition of the predicted ZFPOBI1 binding site, we next cloned short oligonucleotides containing the consensus motif identified by ChIP-seq and motif analysis into the luciferase reporter (Figure 2G). Here too the consensus motif was sufficient to confer ZFPOBI1-dependent repression, whereas repression was lost upon deletion of the KRAB domain. Mutation of the core motif abolished repression, demonstrating that transcriptional repression depends on the integrity of the predicted ZFPOBI1 binding site.

Despite robust FIMO binding site prediction, RLTR10D2 elements display little to no ZFPOBI1 enrichment by ChIP (Figures 2B,C). We therefore also tested the corresponding RLTR10D2 elements, which differs from the RLTR10/RLTR10A/RLTR10D consensus by only two nucleotides (Figures 2E,G). Remarkably, introduction of these two RLTR10D2-specific sequence variants was sufficient to abolish ZFPOBI1-mediated repression in the reporter assay (Figure 2G), providing functional support for the sequence specificity observed in the ChIP-seq data.

Together, these experiments demonstrate that ZFPOBI1 can, in this setting, function as a sequence-specific transcriptional repressor whose activity depends both on an intact DNA-binding motif and on the presence of its KRAB domain, consistent with repression mediated through TRIM28-associated silencing complexes.

### 3.5 Generation of a ZfpObi1 knockout mouse

To assess the transcriptional impact of ZFPOBI1 on RLTR10-driven LIT initiation *in vivo*, we generated a loss-of-function mouse model by deleting exon 4 of *ZfpObi1*, which encodes the zinc finger array, using CRISPR–Cas9 (Figure 1D; Supplementary Figure S3A). Deletion of exon 4 was confirmed by sequencing, and founder animals were crossed to wild-type mice to establish the *ZfpObi1* knockout line (C57BL/6J-Obi1^em1Meschmi/J^). Intercrossing of heterozygous animals yielded viable offspring at expected Mendelian ratios, with both male and female knockout animals appearing phenotypically normal and fertile.

RNA sequencing of *ZfpObi1* knockout oocytes confirmed the absence of the zinc finger–encoding region of exon 4. Instead, aberrant splicing of the mutant allele to a downstream, previously unannotated cryptic exon results in a frameshift and premature stop codon (Supplementary Figure S3A). The predicted protein lacks recognizable domains, including the zinc finger array, and is therefore expected to represent a non-functional ZFPOBI1 variant with impaired DNA-binding capacity (Supplementary Figure S3B).

### 3.6 Loss of ZfpObi1 alters expression of a subset of RLTR10-associated transcripts in oocytes

**FIGURE 3:**
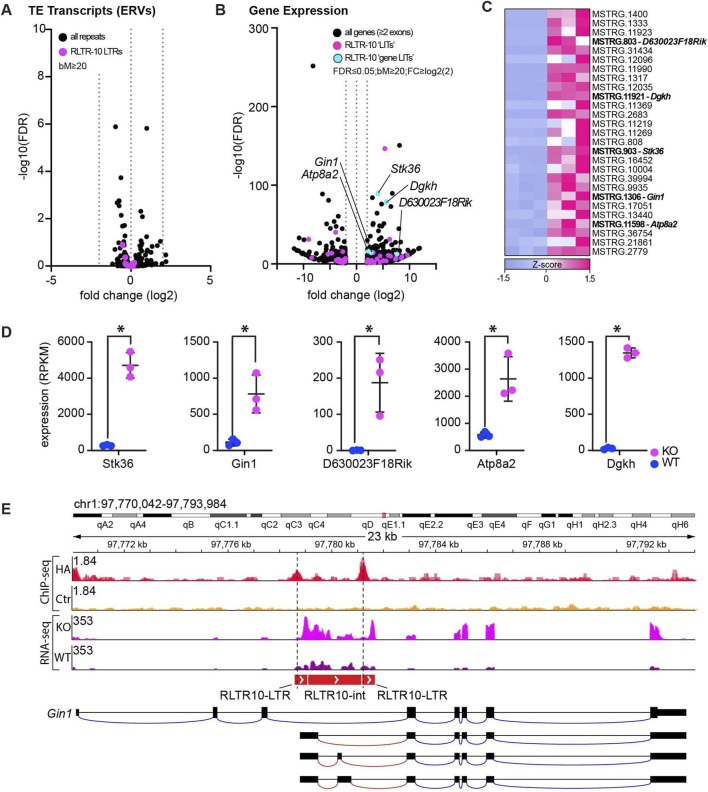
Loss of ZfpObi1 alters expression of a subset of RLTR10-associated transcripts in oocytes. **(A)** Volcano plot showing expression of repetitive element families between wild-type (WT) and *ZfpObi1* knockout (KO) GV oocytes as determined by TEtranscripts. **(B)** Volcano plot of differential expression analysis based on gene-level counts from a *de novo* assembled oocyte transcriptome comparing control (WT) and *ZfpObi1* knockout (KO) GV oocytes. Features associated with RLTR10-derived LTR-initiated transcripts (LITs) are highlighted in magenta, and RLTR10 LIT-associated genes are indicated in cyan. **(C)** Heatmap showing row-wise Z-score–normalized expression of significantly (FDR ≤ 0.05) upregulated RLTR10 LITs identified in **(A)** across WT and KO samples. **(D)** Expression levels (RPKM) of representative genes associated with RLTR10 LITs, illustrating increased expression in KO oocytes relative to WT. **(E)** Genome browser view of the *Gin1* locus showing ZFPOBI1 binding and associated transcription. HA ChIP-seq signal for 3xHA-ZFPOBI1 (red) and control (orange) expressing mESCs is shown. Dashed lines indicate RLTR10-LTR overlapping peaks. RNA-seq signal in *ZfpObi1* knockout (KO) and wild-type (WT) oocytes. Repeat annotation highlighting the RLTR10 full-length element insertion in intron three of *Gin1. De novo* assembled transcripts illustrating endogenous *Gin1* (top) and three RLTR10-driven LTR-initiated transcripts (LITs) splicing into downstream exons of *Gin1*. Splice junctions are indicated in blue (endogenous) or red (LIT associated). **(A–C)** Statistical significance was assessed using DESeq2 (Wald test) and is shown as adjusted P values (FDR). FDR ≤ 0.05 was considered significant (*).

Given the maternal expression of *ZfpObi1* and its association with RLTR10 LTRs, we first assessed global repeat expression using the TEtranscripts pipeline (Jin et al., 2015). Despite the established role of KRAB-ZFPs in transposable element repression, we observed no evidence of global derepression of repeat families in *ZfpObi1*-deficient oocytes (Figure 3A). In particular, RLTR10 LTRs did not show increased expression at the family level, indicating that loss of ZFPOBI1 does not result in a widespread breakdown of TE silencing.

**FIGURE 4:**
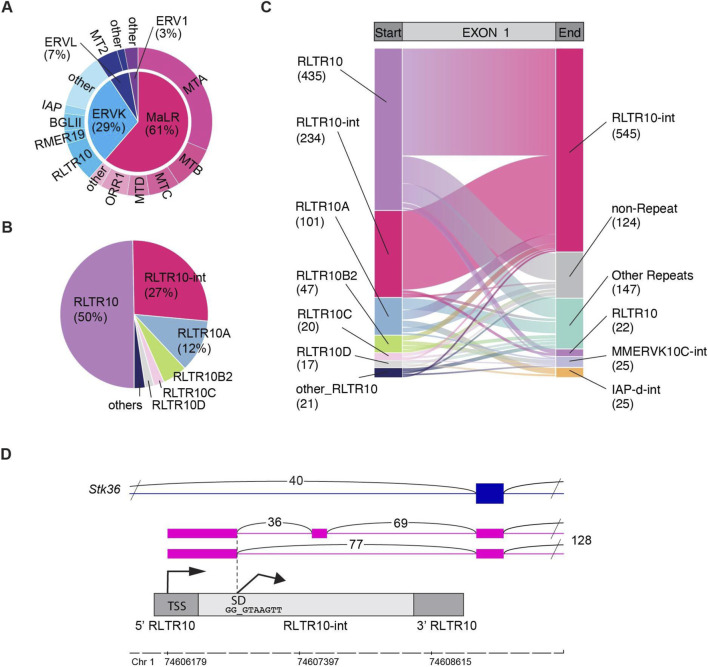
RLTR10 elements contribute to LTR-initiated transcripts and exhibit distinct transcript architectures. **(A)** Distribution of 9,961 LITs based on TSS overlap with RepeatMasker annotations. The majority of LITs originate from MaLR elements (61%), followed by ERVK elements (29%), while ERVL (7%) and ERV1 (3%) contribute minor fractions. **(B)** Subfamily composition of RLTR10-associated LITs (875). RLTR10 LTRs account for 435 events (50%), followed by RLTR10-int elements (27%) and RLTR10A (12%), while RLTR10B2, RLTR10C, RLTR10D, and other subfamilies contribute only minor fractions. **(C)** Sankey-style representation of RLTR10-derived LTR-initiated transcripts (LITs), illustrating connections between repeat families at transcription start sites (left) and the corresponding genomic features at the end of the first exon (right). Flows indicate the number of transcripts linking each initiating RLTR10 subfamily to downstream repeat or non-repeat sequences. The width of each connection is proportional to the number of observed events. **(D)** Representative example of an RLTR10-driven LTR-initiated transcript (LIT) arising from a full-length RLTR10 element. Transcription initiates at the 5′ RLTR10 LTR and extends into the internal (RLTR10-int) region. A splice donor site within the RLTR10-int sequence is used to generate LIT isoforms that splice to downstream regions of *Stk36*. Coordinates correspond to the mm10 genome assembly.

To capture transcript-level changes, we performed a *de novo* transcript assembly of wild-type and knockout GV oocytes combined. Overall LIT distributions remained largely unchanged compared to the control only transcriptome (see below), both at the level of ERV classes and within the RLTR10 family (Figures 4A,B; Supplementary Figures S3C, D; Supplementary Table S16), suggesting that *ZfpObi1* loss does not broadly alter ERV-driven transcriptional initiation, either.

In contrast, gene-level differential expression analysis revealed clear segregation of genotypes (Supplementary Figure S3E; Supplementary Table S17). Focusing on multi-exonic transcripts (≥2 exons) to capture co-option events, we identified 509 differentially expressed genes (baseMean ≥ 20, FDR ≤ 0.05, log_2_FC ≥ 2), comprising 263 upregulated and 247 downregulated genes (Figure 3B, black dots).

Notably, a subset of these genes was associated with RLTR10-derived LITs. Specifically, 28 upregulated and 23 downregulated transcripts were linked to “group a” RLTR10 LITs (Figure 3B, magenta dots). Importantly, five of these represent clear cases of endogenous gene co-option, in which RLTR10-driven LITs splice into downstream genic exons. All five loci exhibit strong transcriptional upregulation in *ZfpObi1*-deficient oocytes (Figure 3B, cyan dots; Figure 3C).

An illustrative example is the *Gin1* locus, where a full-length RLTR10 element inserted within intron three gives rise to multiple LTR-initiated transcripts. These transcripts initiate within the RLTR10 LTR, splice via the internal RLTR10-int region, and join downstream *Gin1* exons, resulting in increased gene expression in the knockout (Figure 3D).

Jointly, these results demonstrate that loss of *ZfpObi1* does not cause widespread derepression of RLTR10 elements but instead affects a limited subset of RLTR10-driven transcripts and associated genes. Given the extensive binding of ZFPOBI1 to RLTR10-derived sequences the limited number of deregulated RLTR10-associated transcripts was somewhat unexpected. To better understand the apparent disconnect between widespread binding and modest transcriptional consequences, we next investigated the overall contribution of RLTR10 elements to LTR-initiated transcript (LIT) formation in oocytes.

### 3.7 RLTR10 elements contribute to LTR-initiated transcripts in oocytes

Endogenous retroviruses (ERVs) contribute to oocyte and early embryonic transcriptomes through transcriptional co-option, whereby LTRs can function as alternative promoters to generate chimeric transcripts. In oocytes, this process gives rise to LTR-initiated transcripts (LITs) that extend into downstream genomic regions (Peaston et al., 2004; Veselovska et al., 2015; Franke et al., 2017; Brind’Amour et al., 2018). While MaLR elements are known to be active in oocytes and MERVL elements drive transcription at the 2-cell stage (Burton and Torres-Padilla, 2025), the contribution of RLTR10 elements to LIT formation has not been systematically characterized. To address this, we reprocessed published oocyte RNA-seq datasets (Seah et al., 2025) and performed *de novo* transcript assembly. LTR-initiated transcripts were defined by intersecting transcription start sites (TSSs), collapsing transcripts with identical first exons, with ERV annotations from RepeatMasker (n = 9,961 transcripts) (Supplementary Table S15). The majority of LITs originate from MaLR elements (61%; 6,103/9,961), followed by ERVK elements (29%; 2,913/9,961), while ERVL and ERV1 families contribute only minor fractions (Figure 4A; Supplementary Table S15). Among MaLR-derived LITs, MTA-family elements account for the largest proportion of initiation events, consistent with previous reports (Peaston et al., 2004; Veselovska et al., 2015; Franke et al., 2017; Brind’Amour et al., 2018). Within the ERVK class, RLTR10 elements dominate LIT initiation (Figure 4A). Of the 875 RLTR10-derived LITs, 88% (773/875) originate from “group a” RLTR10 elements, including both LTR and internal (RLTR10-int) sequences. RLTR10 LTRs alone account for approximately half of all RLTR10-driven LITs (435/875), with additional contributions from RLTR10-int elements (27%; 234/875) and RLTR10A LTRs (12%; 101/875). Other RLTR10 subfamilies, including RLTR10D2 LTRs, contribute only minor fractions (Figure 4B; Supplementary Table S13).

### 3.8 RLTR10 elements separate transcription initiation and splicing functions

The strong contribution of RLTR10 elements to oocyte LIT formation prompted us to examine their transcript structure and splicing patterns in detail. MaLR-driven LITs, including those derived from MTA elements, involve both transcriptional initiation and splice donor usage within the LTR itself. Consequently, MTA LTRs in both solo and internal-associated configurations can independently support LIT formation (Franke et al., 2017) (Supplementary Figure S4A). To investigate RLTR10-driven LITs, we annotated transcription start sites (TSSs) together with first exon–intron boundaries (splice donor sites) for all assembled transcripts (Figure 4C; Supplementary Table S13). Strikingly, RLTR10-derived LITs exhibit a distinct architecture: while transcription is typically initiated within the LTR, splice donor sites are rarely located within the LTR itself. Instead, first exons frequently extend into downstream internal (-int) regions, which harbour the predominant splice donor sites used to connect to downstream exons (Figure 4C). Quantitatively, 60% (316/536) of RLTR10- and RLTR10A-LTR–driven LITs splice from adjacent RLTR10-int regions into downstream exons, whereas 37% (200/536) splice from other downstream sequences, including genomic regions or other repeat elements. Only 4% (23/536) utilize splice donor sites within the LTR itself (Figure 4C). Notably, 27% of RLTR10-associated LITs were annotated as initiating within RLTR10-int regions (Figures 4B,C). Given that internal ERV sequences typically lack promoter activity, this likely reflects incomplete recovery of transcript 5′ ends or assembly bias associated with Smart-seq2. Supporting this interpretation, 95% of these transcripts are preceded by an RLTR10 family LTR within 1 kb, most commonly RLTR10-LTRs located within 20 bp upstream of the annotated TSS (Supplementary Figures S4B, C), suggesting that transcription in these cases also originates from upstream LTRs. An illustrative example of this architecture is an oocyte-specific alternative transcript of the serine/threonine kinase *Stk36,* which is also significantly deregulated in *ZfpObi1* KO oocytes (Figures 3B–D). Here, transcription initiates within the 5′ RLTR10 LTR of a full-length ERV insertion located in intron 5 and extends into the internal RLTR10-int region, from which a canonical splice donor site is used to connect to downstream exons (Figure 4D).

Altogether, these findings reveal a distinct mode of ERV-driven transcription in which RLTR10 elements separate transcription initiation and splicing functions across LTR and internal sequences. This contrasts with MaLR-derived LITs (Franke et al., 2017) and highlights a structural specialization of RLTR10 elements in the oocyte transcriptome. Given the apparent importance of internal ERV sequences for RLTR10-driven LIT formation, we next asked how frequently these structural features are retained across genomic RLTR10 insertions and whether differences in ERV architecture might help explain the selective transcriptional activity of only a subset of RLTR10 elements.

### 3.9 Structural organization of “group a” RLTR10 LTRs in the mouse genome

RLTR10 LTR subfamilies constitute a highly abundant class of ERVK elements in the mouse genome and are present either as solo LTRs or in association with internal ERV sequences (Jurka et al., 2005; Kawase and Ichiyanagi, 2023). To further characterize the structural context of ZFPOBI1-associated “group a” LTRs, we first examined their association with internal ERV sequences (*-int). RLTR10- and RLTR10A-LTRs are most frequently found in proximity to RLTR10-int elements, accounting for 45% and 23% of insertions, respectively (Supplementary Figures S5A, B). In contrast, RLTR10D-LTRs are more commonly associated with IAP-d internal sequences (45%), consistent with previous reports (Supplementary Figure S5C) (Kawase and Ichiyanagi, 2023).

Given that structural context may influence both regulatory activity and KRAB-ZFP targeting, we next stratified “group a” RLTR10 LTRs into solo elements and those associated with internal ERV sequences. We defined LTRs as “associated” if they were located within 50 bp of a cognate internal (*-int) element and part of a consistent ERV configuration (LTR–int–LTR) of the same subfamily. LTRs lacking such association were classified as “solo elements” (Supplementary Tables S8–S14). Using these criteria, 38% (1,026/2,702) of RLTR10-LTRs were classified as “solo elements,” whereas 53% (1422/2,702) were “associated” with internal sequences (Supplementary Figure S5D). The remaining 9% of RLTR10 LTRs were found in fragmented/partial ERV configuration and excluded from analysis. RLTR10A-LTRs were predominantly found as “solo elements” (77%; 599/773) (Supplementary Figure S5E). 30% (144/485) RLTR10D-LTRs were “associated” with IAP-d-int elements while 55% (268/485) were present as “solo LTRs” (Supplementary Figure S5F).

Because RLTR10-driven LIT formation predominantly relies on internal ERV sequences, we next asked whether full-length RLTR10 elements differ from solo LTRs with respect to KRAB-ZFP targeting and TRIM28 recruitment.

### 3.10 ZFPOBI1/TRIM28 interaction with “group a” RLTR10 solo LTRs in mESCs

**FIGURE 5:**
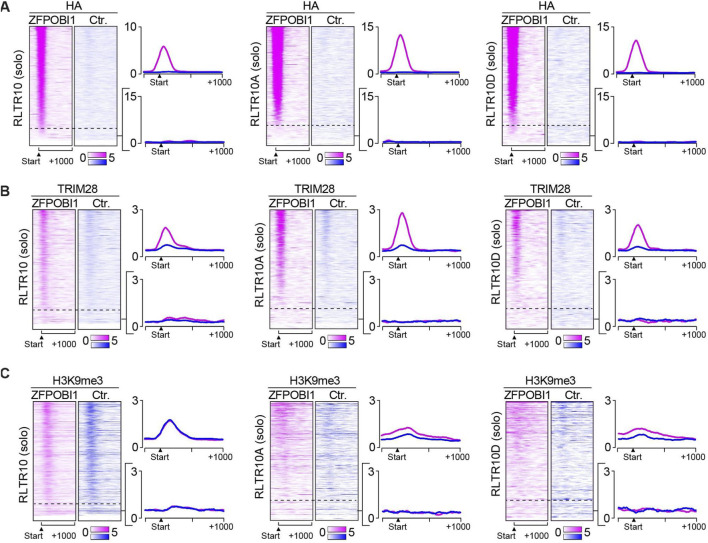
ZFPOBI1-dependent TRIM28 recruitment at RLTR10 solo LTRs. **(A)** HA-ChIP enrichment of ZFPOBI1 across solo LTRs of the indicated RLTR10 subfamilies (RLTR10, RLTR10A, RLTR10D), shown as heatmaps centred on LTR start sites (±1 kb) with corresponding average profiles. Regions are stratified based on ZFPOBI1 enrichment (upper panels) or absence of enrichment (lower panels). Strong and specific enrichment is observed at ZFPOBI1-bound loci compared to control. **(B)** TRIM28 ChIP-seq signal across the same regions, displayed with identical stratification. TRIM28 enrichment is preferentially observed at ZFPOBI1-bound loci and is reduced at loci lacking ZFPOBI1 binding. **(C)** H3K9me3 ChIP-seq signal across the same regions shows a similar pattern, with higher levels at ZFPOBI1-bound loci compared to unbound loci. Baseline H3K9me3 levels vary between subfamilies, with RLTR10 elements displaying elevated signal in control conditions relative to RLTR10A and RLTR10D. (Control [Ctr.] refers to “empty expression vector transfected” mESCs).

Lacking the means to conduct binding studies directly in oocytes and embryos, we used control and 3xHA-ZFPOBI1-overexpressing mESCs to investigate genomic binding and TRIM28 recruitment. HA signal is highly enriched at most, but not all, RLTR10, RLTR10A, and RLTR10D solo LTRs, consistent with the high incidence of the predicted binding motif within these “group a” RLTR10*-LTRs (Figure 5A). ZFPOBI1 binding is accompanied by robust recruitment of TRIM28, providing further evidence for a functional ZFPOBI1–TRIM28 interaction. Notably, little to no TRIM28 enrichment is observed at these solo LTR elements in control mESCs (Figure 5B). Consistent with this observation, genome-wide comparison of ZFPOBI1 and TRIM28 ChIP-seq datasets revealed only limited overlap between both factors in control mESCs, whereas extensive overlap was observed following ZFPOBI1 expression (Supplementary Figure S5G). Examination of RLTR10-associated TRIM28 peaks further revealed a redistribution of TRIM28 occupancy from predominantly RLTR10-int elements towards RLTR10 and RLTR10A LTRs upon ZFPOBI1 expression, supporting ZFPOBI1-dependent recruitment of TRIM28 to group a RLTR10 LTRs (Supplementary Figure S5H). The subset of solo RLTR10, RLTR10A, and RLTR10D elements that do not exhibit ZFPOBI1 binding also lack detectable TRIM28 recruitment (Figures 5A,B, lower panels). Likewise, the ZFPOBI1-independent “group b” family members RLTR10E and RLTR10F show little to no ZFPOBI1 enrichment and no detectable increase in TRIM28 recruitment above endogenous levels (Supplementary Figures S5I, J). These observations support the specificity of ZFPOBI1-dependent TRIM28 recruitment to the bound subset of “group a” RLTR10 elements.

Changes in H3K9me3 are considerably more subtle than those observed for TRIM28 recruitment. While RLTR10A and RLTR10D elements display modest increases in H3K9me3 signal upon ZFPOBI1 expression, RLTR10 solo LTRs already exhibit substantial H3K9me3 enrichment in control mESCs and show little additional increase following ZFPOBI1 overexpression (Figure 5C). This indicates that ZFPOBI1-dependent TRIM28 recruitment is not necessarily accompanied by substantial *de novo* H3K9me3 deposition and suggests that many RLTR10-derived loci reside within a pre-existing repressive chromatin environment in mESCs.

### 3.11 Full-length RLTR10 elements exhibit composite KRAB-ZFP targeting

**FIGURE 6:**
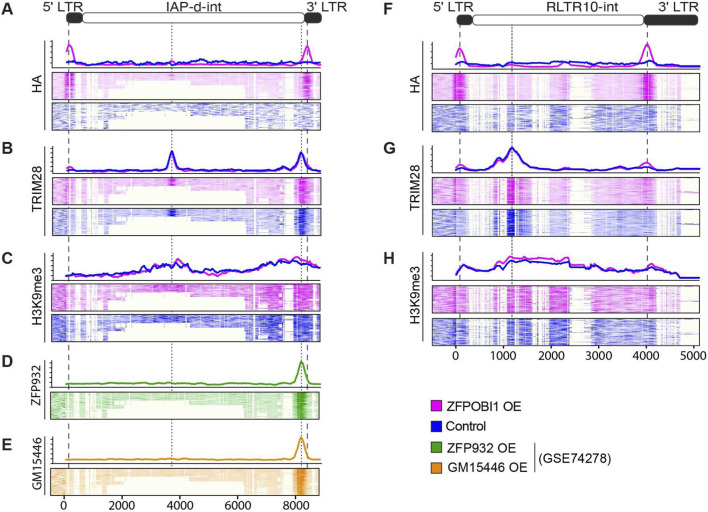
Full-length RLTR10 elements exhibit composite KRAB-ZFP targeting. ChIP-seq metaprofiles (MSA-based enrichment plots) across full-length RLTR10D/Iap-d-int/RLTR10D elements aligned from the 5′ LTR through the internal region (IAP-D-int) to the 3′ LTR. **(A–C)** ZFPOBI1 overexpression (magenta) and control (blue) signals are shown as average profiles with corresponding heatmaps. **(A)** HA-ChIP of ZFPOBI1 shows strong enrichment at both LTRs, with minimal signal across the internal region. **(B)** TRIM28 is enriched at LTRs and additionally across the internal region, indicating recruitment from multiple targeting sites. **(C)** H3K9me3 forms a broad domain spanning the entire element, consistent with spreading from nucleation sites. **(D, E)** ChIP-seq profiles of ZFP932 (D, green) and GM15446 (E, orange) show enrichment at the internal IAP-D sequence near the 3′ LTR–internal boundary, distinct from ZFPOBI1 binding at LTRs. **(F–H)** ChIP-seq metaprofiles across RLTR10 elements associated with RLTR10-int sequences, displayed as in **(A–C)**. **(F)** ZFPOBI1 remains enriched at LTRs, whereas **(G)** TRIM28 and **(H)** H3K9me3 extend across the internal region, mirroring the pattern observed at RLTR10D/IAP-D elements. This indicates that full-length ERVs are targeted by multiple KRAB-ZFPs recognizing adjacent sequence features in LTR and internal regions, resulting in integrated TRIM28 recruitment and formation of extended H3K9me3 domains. (Control [Ctr.] refers to “empty expression vector transfected” mESCs).

We identified 72 RLTR10D–IAP-d-int–RLTR10D elements matching our curation criteria. To examine binding and chromatin features across these loci, we generated multiple sequence alignment (MSA)-based coverage plots for 3xHA-ZFPOBI1, TRIM28, and H3K9me3 (Figures 6A–C). ZFPOBI1 binding was detected at the 5′ regions of both LTRs in 3xHA-ZFPOBI1-overexpressing mESCs, but not in control cells, accompanied by a mild increase in TRIM28 recruitment (Figure 6B, dashed lines), mirroring observations at the solo LTRs (Figure 5A, dashed lines). ZFPOBI1-independent TRIM28 enrichment was observed at two additional sites within the IAP-d-int region (mid and distal 3′ region), present in both control and ZFPOBI1-overexpressing cells (Figure 6B, dotted lines). Consistent with this, H3K9me3 enrichment across the locus is largely dominated by these endogenous TRIM28-bound regions (Figure 6C). Previous work has shown that ZFP932 and its paralog GM15446 target IAP-d-int elements at the 3′ boundary of the internal region (Ecco et al., 2016). This coincides with the TRIM28 enrichment observed at this site in our data (Figures 6B,D,E), supporting the notion that these factors mediate TRIM28 recruitment independently of ZFPOBI1. The KRAB-ZFP responsible for TRIM28 recruitment at the mid-region of the IAP-d-int element remains unknown.

RLTR10–RLTR10-int–RLTR10 elements are substantially more abundant in the mouse genome, with 711 loci passing our selection criteria. MSA-based coverage plots reveal ZFPOBI1 binding at both LTRs under overexpression conditions, with signal localized to the 5′ regions of each LTR, consistent with the motif distribution (Figure 6F). This is accompanied by a modest increase in TRIM28 recruitment at these sites, which is absent in control mESCs (Figure 6G, dashed lines). In contrast, RLTR10-int regions display strong TRIM28 enrichment in both control and ZFPOBI1-overexpressing cells, indicating ZFPOBI1-independent recruitment (Figure 6G, dotted lines). As observed for IAP-d-int elements, this endogenous TRIM28 binding accounts for the majority of H3K9me3 signal across the locus (Figure 6H, dotted lines). A similar pattern is observed for the smaller set of RLTR10A–RLTR10-int–RLTR10A elements (n = 12), indicating that this organization is conserved across RLTR10 subfamilies.

These findings reveal a modular pattern of KRAB-ZFP targeting across full-length ERV elements, in which distinct proteins can independently recognize LTR and internal sequences. This supports a model in which multiple KRAB-ZFPs contribute independently to TRIM28 recruitment across different regions of the same ERV insertion. Such combinatorial targeting may enhance the robustness of repression by providing multiple recruitment platforms for TRIM28-containing silencing complexes.

## 4 Discussion

Here we identify the mouse KRAB-ZFP ZFPOBI1 as a maternally expressed factor whose binding specificity is directed towards RLTR10-derived sequences. Using heterologous target-identification and reporter assays, we show that ZFPOBI1 preferentially recognizes RLTR10, RLTR10A, and RLTR10D elements and can recruit the co-repressor TRIM28 to these sites. In the physiological context of the oocyte, loss of *ZfpObi1* is associated with modest deregulation of a subset of RLTR10-derived LTR-initiated transcripts (LITs), suggesting a role in the regulation of RLTR10-associated transcriptional activity.

**FIGURE 7:**
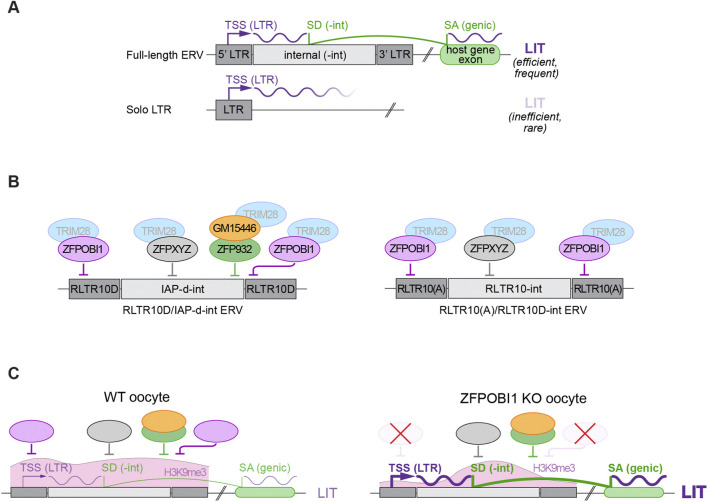
Model for RLTR10 transcriptional co-option and multilayered KRAB-ZFP targeting. **(A)** ERV structural configuration and transcriptional co-option. Full-length RLTR10 elements support efficient LTR-initiated transcript (LIT) formation, with transcription initiating at the 5′ LTR (TSS) and splicing occurring via donor sites within internal (-int) regions to downstream genic splice acceptors. In contrast, solo LTRs lack efficient splice donor sites, resulting in rare or inefficient LIT formation. **(B)** Composite KRAB-ZFP targeting of ERVs. Distinct KRAB-ZFPs target different regions of full-length ERVs. ZFPOBI1 binds RLTR10 LTRs, whereas additional KRAB-ZFPs (e.g., ZFP932 and GM15446 for IAP-d-int elements, and other factors for RLTR10-int regions) target internal sequences. This modular targeting results in composite recruitment of the co-repressor TRIM28 across ERV loci. **(C)** Effect of *ZfpObi1* loss on RLTR10-driven transcription. In wild-type oocytes, multilayered KRAB-ZFP targeting establishes H3K9me3 and represses ERV-driven transcription. Upon maternal loss of *ZfpObi1*, repression at LTRs is partially reduced, while internal targeting remains intact, resulting in selective upregulation of a subset of RLTR10-driven LITs.

A central observation emerging from our study is that RLTR10-driven LTR-initiated transcripts (LITs) exhibit a distinct architectural organization compared to, for example, previously described MaLR-derived transcripts. While MaLR elements frequently provide both promoter activity and splice donor sites within the LTR itself (Franke et al., 2017), RLTR10 elements appear to partition these functions across different regions of the ERV. Specifically, RLTR10 LTRs predominantly act as promoter and transcriptional start sites, whereas splice donor usage is preferentially derived from downstream internal (-int) sequences. This separation of transcription initiation and RNA processing functions suggests that ERV structural organization is an important determinant of transcriptional co-option (Figure 7A). Consistent with this model, our analyses indicate that RLTR10-driven LIT formation is strongly associated with internal sequence context, whereas solo LTRs contribute only infrequently as transcriptional initiators. Thus, the regulatory potential of RLTR10 elements is not solely encoded within the LTR but depends on the presence of associated internal sequences. Such structural dependence likely contributes to the selective activation of only a subset of genomic RLTR10 elements in oocytes.

Interestingly, this structural organization is mirrored by the pattern of KRAB-ZFP targeting across full-length RLTR10-derived ERVs. ZFPOBI1 binding is restricted to LTR sequences, whereas internal regions are independently targeted by additional KRAB-ZFPs, including ZFP932 and GM15446 at IAP-d-int elements, and likely further, yet unidentified factors at RLTR10-int regions (Figure 7B). These observations suggest that multiple KRAB-ZFPs can independently recruit TRIM28 to different regions of the same ERV insertion, creating a modular pattern of silencing complex recruitment across full-length elements.

In line with this, both *ZfpObi1* and *GM15446* are among the most highly expressed KRAB-ZFPs in oocytes (top 10), and *Zfp932* is also abundantly expressed (rank 24). The presence of multiple highly expressed KRAB-ZFPs targeting distinct regions of ERVs provides a potential mechanistic basis for the robustness of ERV repression in oocytes and is consistent with a model of multilayered ERV targeting.

Despite clear ZFPOBI1-dependent recruitment of TRIM28 to RLTR10-derived sequences, loss of *ZfpObi1* does not result in widespread transcriptional deregulation. Instead, transcriptional effects are limited to a subset of RLTR10-driven LITs and associated genes (Figure 7C). This observation is consistent with both the limited capacity of solo LTRs to drive LIT formation and the extensive functional redundancy among KRAB-ZFPs, a hallmark of the evolutionary arms race between transposable elements and their repressors (Imbeault et al., 2017; Bruno et al., 2019; Wells and Feschotte, 2020; Kosuge et al., 2024). Recent genome-wide analyses further suggest that many ERV families undergo repeated cycles of KRAB-ZFP-mediated repression, escape, and retargeting by additional KRAB-ZFPs, resulting in multilayered and overlapping repression systems (Davis et al., 2026). Redundant targeting of ERVs at both LTR and internal regions may therefore buffer the transcriptional consequences of losing individual KRAB-ZFPs, such as *ZfpObi1*.

More broadly, our findings align with models in which KRAB-ZFP/ERV interactions are shaped by co-evolutionary dynamics, leading to subfamily-specific diversification of both binding specificity and regulatory function. In this context, sequence divergence within RLTR10 elements likely underlies selective ZFPOBI1 targeting, generating distinct subsets of elements that differ in their responsiveness to ZFPOBI1-mediated regulation.

Finally, although *ZfpObi1* knockout mice appear viable and fertile under standard laboratory conditions, the observed transcriptional changes raise the possibility that subtle phenotypic consequences may emerge under stress conditions, during aging, or across generations. Given the role of ERVs in genome regulation and transcriptome diversification, it will be important to investigate whether disruption of multilayered repression mechanisms influences epigenetic inheritance, genome integrity, or developmental robustness in more sensitive contexts.

In conclusion, our findings highlight the close relationship between ERV structure, transcriptional co-option, and KRAB-ZFP targeting. In oocytes, where extensive epigenetic reprogramming creates a permissive transcriptional environment, full-length RLTR10 elements appear particularly well suited to contribute to transcriptome diversification while remaining subject to multilayered regulation by multiple KRAB-ZFPs.

## Data Availability

The datasets presented in this study can be found in online repositories. The names of the repository/repositories and accession number(s) can be found in the article/Supplementary Material.
