# Supplementary figures and images for: The maternal KRAB-ZFP ZFPOBI1 reveals structural constraints governing ERV transcriptional co-option in mouse oocytes
Source: Front Cell Dev Biol. 2026 Jul 14;14:1851851. doi: 10.3389/fcell.2026.1851851 (PMC13408016; doi:10.3389/fcell.2026.1851851)

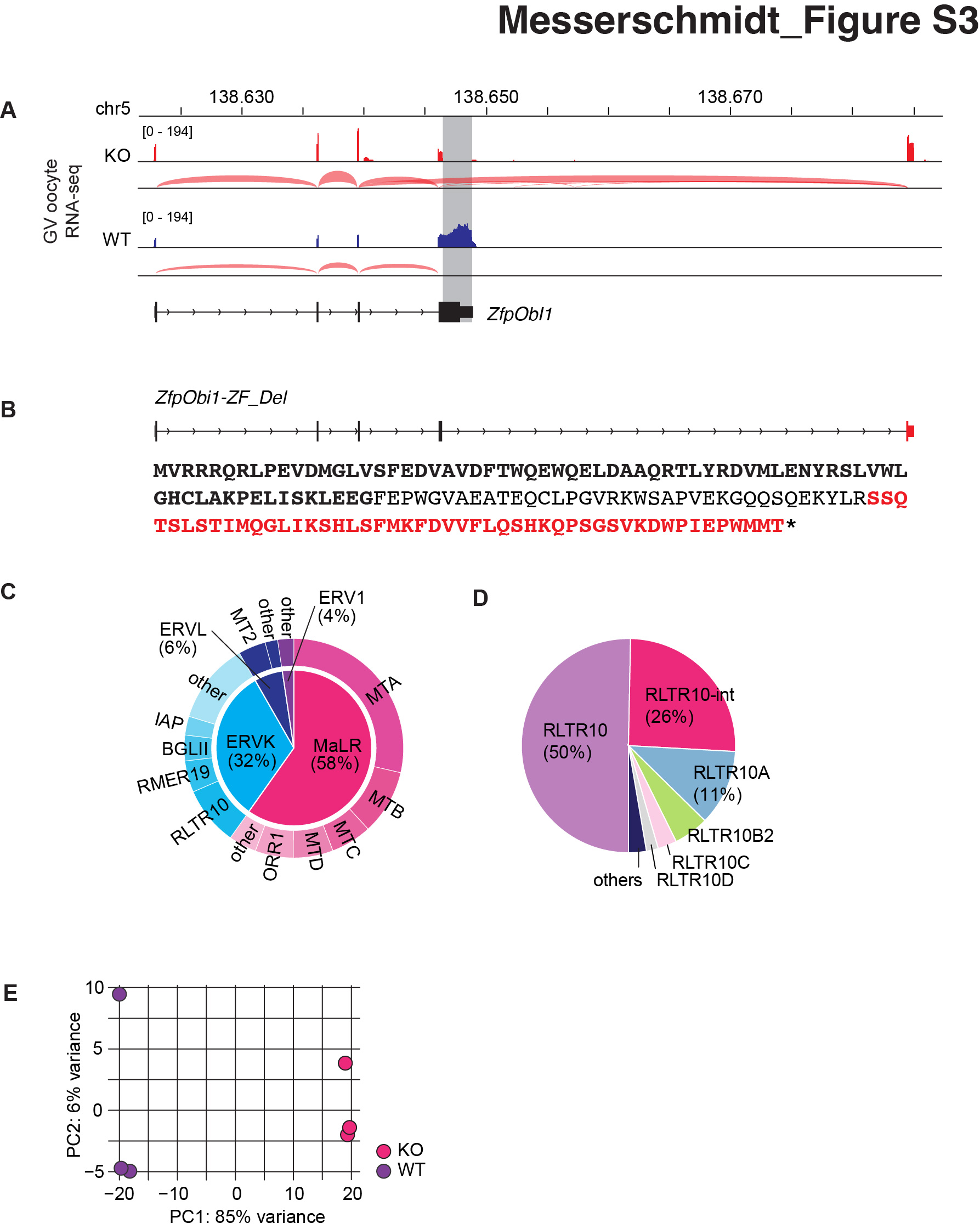

Supplement: Supplementary file 1 [file Image3.jpeg]

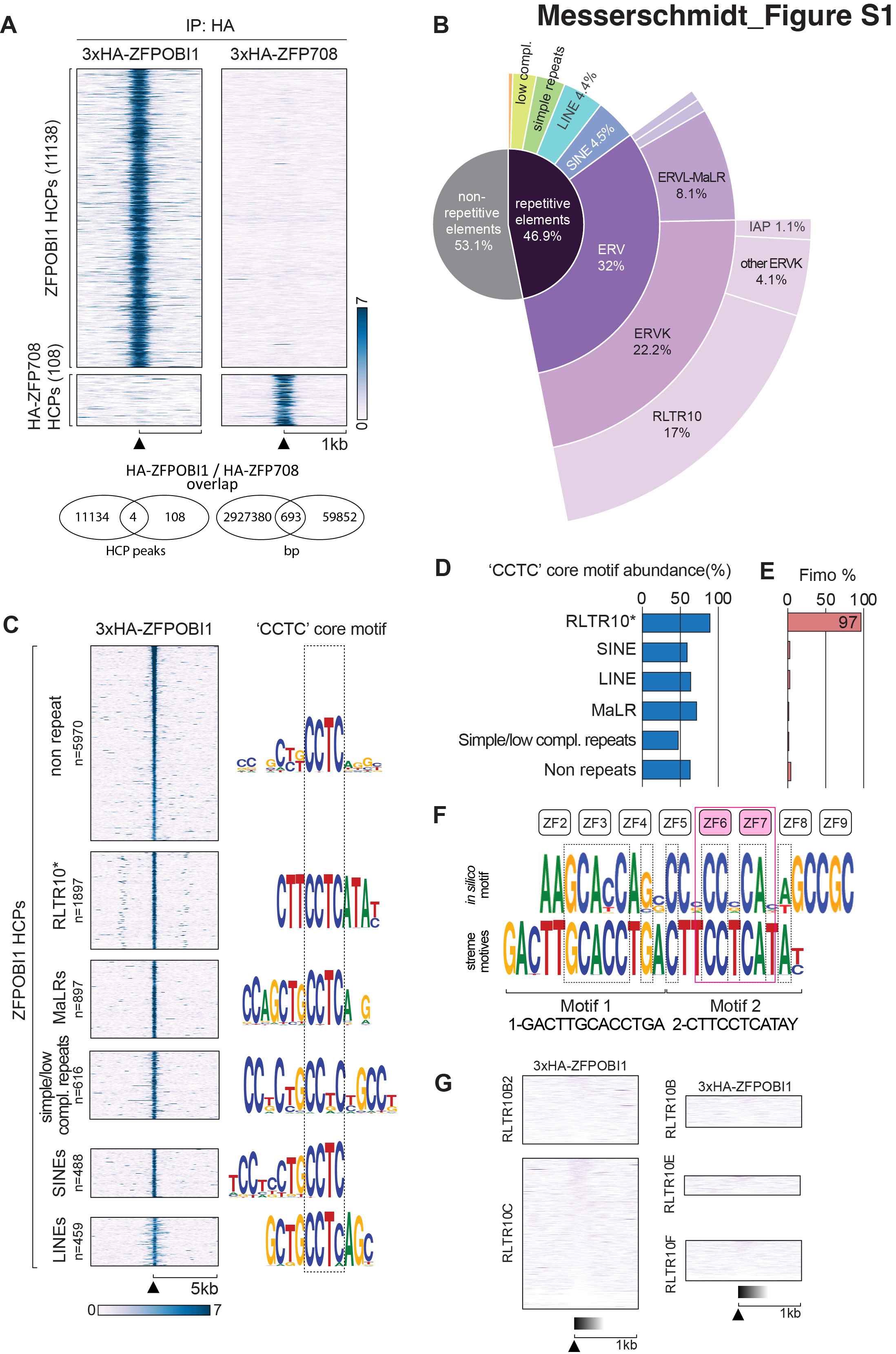

Supplement: Supplementary file 3 [file Image1.jpeg]

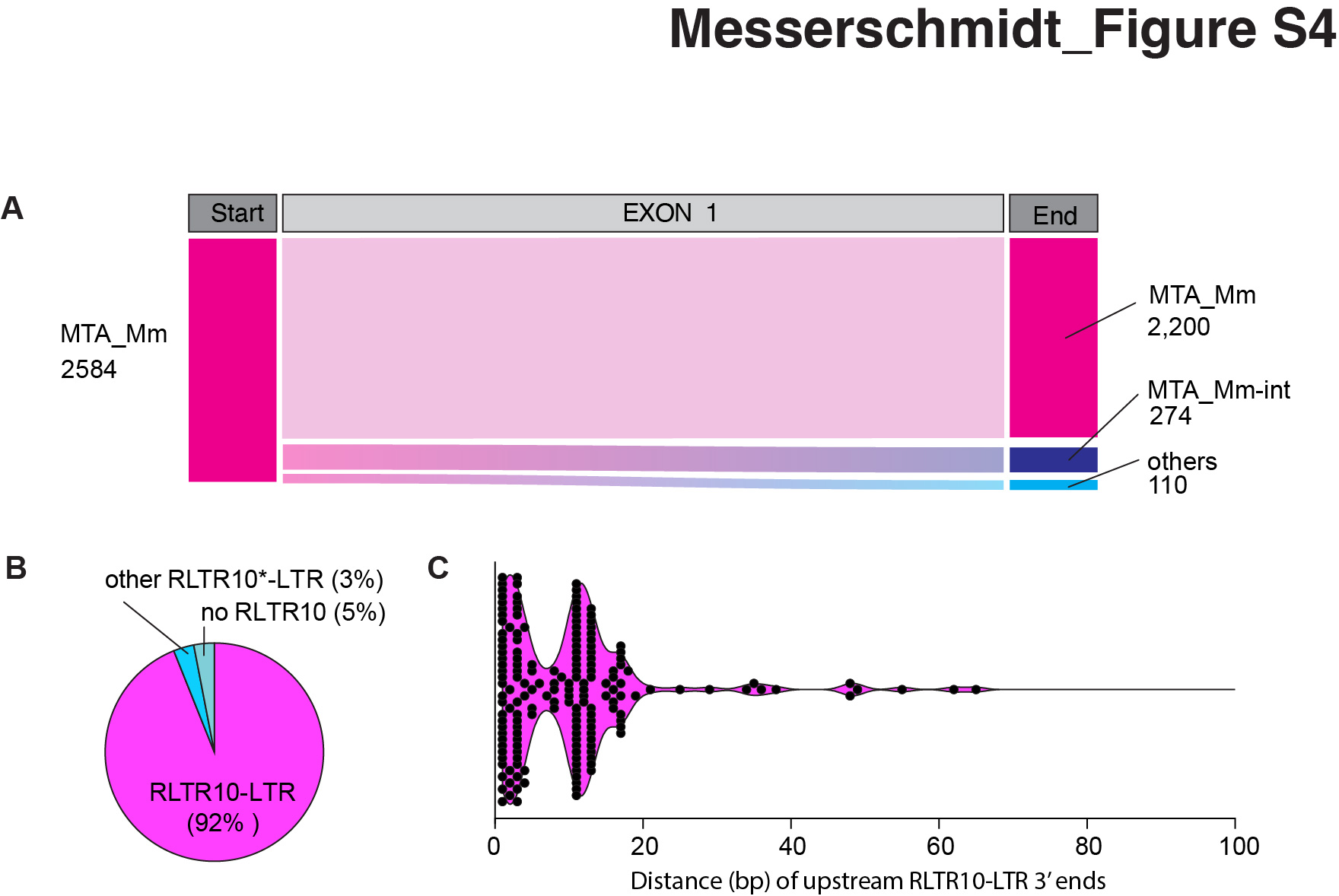

Supplement: Supplementary file 4 [file Image4.jpeg]

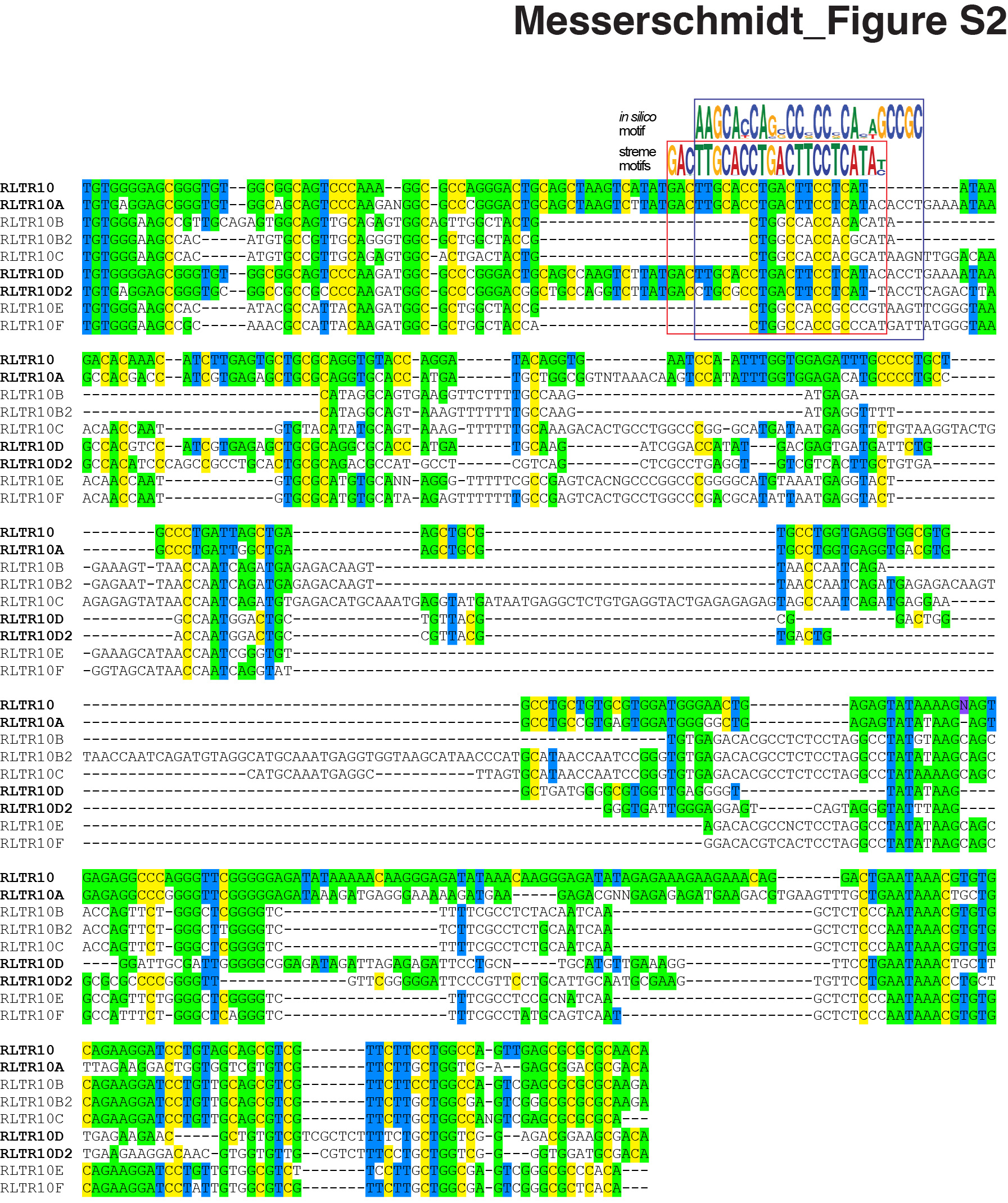

Supplement: Supplementary file 5 [file Image2.jpeg]

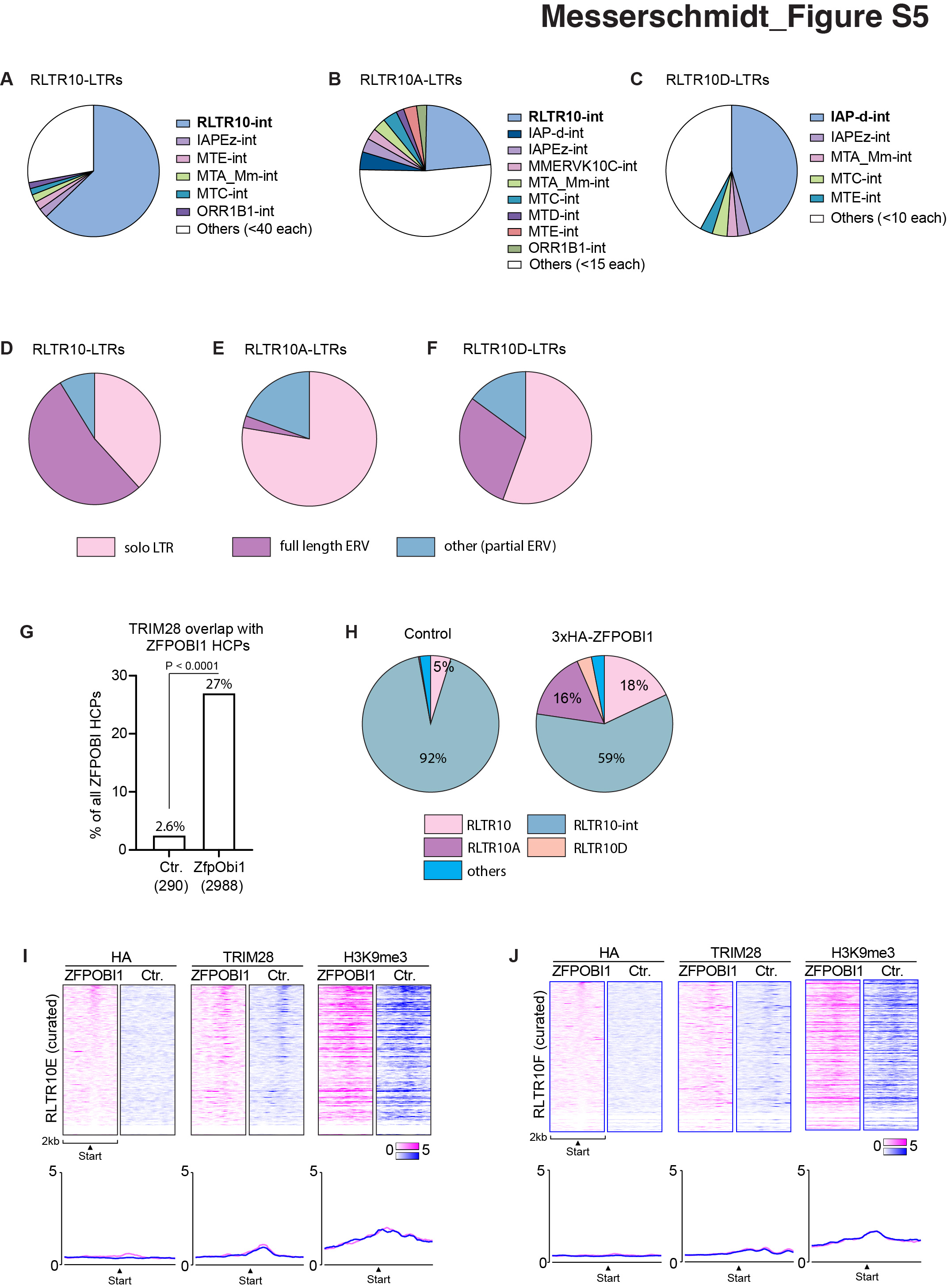

Supplement: Supplementary file 6 [file Image5.jpeg]
