# Supplementary material for: The maternal KRAB-ZFP ZFPOBI1 reveals structural constraints governing ERV transcriptional co-option in mouse oocytes
Source: Front Cell Dev Biol. 2026 Jul 14;14:1851851. doi: 10.3389/fcell.2026.1851851 (PMC13408016; doi:10.3389/fcell.2026.1851851)
Supplement: Supplementary file 2 [file Supplementaryfile1.docx]

**Supplementary figures legends**

**Figure S1. ZFPOBI1 exhibits highly specific binding to RLTR10-derived sequences (A)** HA-ChIP enrichment of ZFPOBI1 and ZFP708 plotted over their respective target regions reveals strong signal restricted to cognate targets, indicating high specificity of each ChIP experiment. Direct comparison of ZFPOBI1 and ZFP708 peak sets reveals minimal overlap, with only four shared regions (~700 bp total overlap), demonstrating largely distinct genomic binding profiles despite their shared KRAB-ZFP architecture. **(B)** Sunburst plot illustrating that ZFPOBI1 ChIP–seq peaks are enriched within repetitive elements, particularly ERV families, with hierarchical breakdown into classes and subfamilies. **(C)** ChIP–seq enrichment of 3xHA-ZFPOBI1 across the six most prominent HCP subcategories, shown as heatmaps centred on peak summits, alongside the top enriched motifs identified by STREME for each category. A shared core motif (CCTC) is highlighted. **(D)** Abundance of the CCTC core motif across major genomic categories underlying HCPs. **(E)** Fraction of elements containing a second RLTR10-associated motif identified by FIMO. While the core motif is broadly distributed, the RLTR10-specific motif is strongly enriched in RLTR10 elements. **(F)** Alignment of the predicted ZFPOBI1 DNA-binding motif, derived from zinc finger (ZF) domain composition, with motifs identified by STREME. Individual zinc fingers are indicated above, showing strong agreement between predicted DNA-contacting residues and experimentally derived motifs, with ZF6–7 corresponding to the core motif region. **(G)** Heatmaps showing ZFPOBI1 ChIP-seq signal across curated RLTR10 ‘group b’ elements and individual subfamilies, aligned at the 5′ LTR boundary (±1 kb). In contrast to the RLTR10 ‘group a’ subfamilies shown in Figure 2D, little to no enrichment is observed across RLTR10B, RLTR10C, RLTR10E and RLTR10F.

**Figure S2** **Sequence alignment of RLTR10 subfamily consensus sequences highlights divergence at the ZFPOBI1 binding site.** Multiple sequence alignment of representative RLTR10 subfamily consensus sequences (group a and group b) is shown, with nucleotides coloured by identity. The region corresponding to the ZFPOBI1 binding site is highlighted (red box), with the *in silico*-derived motif shown above (blue box). ‘Group a’ elements (in bold) display strong conservation of the binding motif, whereas ‘group b’ elements exhibit substantial sequence divergence within the motif.

**Figure S3 Generation and transcriptomic characterization of the ZfpObi1 knockout mouse (A)** CRISPR-mediated deletion of *ZfpObi1* results in altered transcript structure in GV oocytes. Genome browser view of RNA-seq coverage and splice junctions across the *ZfpObi1* locus in wild-type (WT) and knockout (KO) GV oocytes. The targeted region (grey shading), corresponding to exon 4 encoding the zinc finger domains, shows strong read coverage in WT but is absent in KO samples, consistent with successful deletion. In KO oocytes, splice junctions bypass the deleted region and instead connect to a downstream alternative exon, indicating altered transcript processing following the deletion. Gene annotation is shown below (mm10). **(B)** Schematic depiction and amino acid sequence of the putative protein derived from the CRISPR-targeted *ZfpObi1* locus. Zinc finger (ZF) deletion in exon 4 yields a KRAB-containing (bold) predicted truncated protein with a frameshift-derived non-native tail (red) and early stop. **(C)** Distribution of LITs based on TSS overlap with RepeatMasker annotations identified in WT and *ZfpObi1*-KO GV oocyte transcriptomes. The majority of LITs originate from MaLR elements (60%), followed by ERVK elements (32%), while ERVL (6%) and ERV1 (2%) contribute minor fractions. (D) Subfamily composition of RLTR10-associated LITs. RLTR10 LTRs account for 50%, followed by RLTR10-int elements (26%) and RLTR10A (11%), while RLTR10B2, RLTR10C, RLTR10D, and other subfamilies contribute only minor fractions.

**Figure S4 Splicing patterns of MTA-derived LTR-initiated transcripts (LITs) in mouse oocytes** **(A)** Schematic overview of transcription initiation and splicing behaviour of MTA-derived LITs. Transcripts initiate within MTA_Mm LTRs and predominantly splice within the same element, while only a small subset extend into downstream internal regions (MTA_Mm-int) or splice into downstream repeat elements or non-repetitive genomic regions. **(B)** Distribution of upstream RLTR10*-LTR elements located within 1 kb of RLTR10-int–associated LITs. Most loci are associated with RLTR10-LTRs, while only a small fraction map to other RLTR10 subfamilies or lack a nearby RLTR10 element. **(C)** Distribution of distances between RLTR10-int LITs and upstream RLTR10 LTR 3′ ends. Distances are shown for RLTR10-associated loci within a 0-100 bp window (88% of all).

**Figure S5. Structural organization of RLTR10 subfamilies and control analyses of ZFPOBI1-independent RLTR10 elements.** **(A–C)** Composition of internal ERV annotations in proximity to RLTR10 LTR ‘group a’ subfamilies. Each LTR was assigned to the nearest annotated internal ERV sequence without consideration of distance or strand orientation. RLTR10 LTRs **(A)** and RLTR10A LTRs **(B)** predominantly associate with RLTR10-int elements, whereas RLTR10D LTRs **(C)** are enriched for IAP-d internal sequences. ‘Others’ represents all internal ERV sequences occurring fewer than 40 (RLTR10), 15 (RLTR10A), or 10 (RLTR10D) times. **(D–F)** Pie charts showing the proportion of RLTR10 **(D)**, RLTR10A **(E)**, and RLTR10D LTRs **(F)** classified as solo LTRs, full-length ERVs (LTR–internal–LTR), or others (partial ERV insertions). While RLTR10A elements are predominantly found as solo LTRs, RLTR10 and RLTR10D subfamilies exhibit a higher proportion of full-length ERV configurations, indicating subfamily-specific differences in structural integrity and genomic context. **(G)** Percentage of ZFPOBI1 high-confidence peaks (HCPs) overlapping TRIM28 ChIP-seq peaks in control and 3xHA-ZFPOBI1-expressing mESCs. Numbers in parentheses indicate the number of overlapping regions. TRIM28 overlap with ZFPOBI1-bound loci increases markedly following ZFPOBI1 expression. Statistical significance was assessed by Fisher's exact test (*P < 0.0001). **(H)** Distribution of RLTR10-family annotations associated with TRIM28 peaks overlapping ZFPOBI1 HCPs in control and 3xHA-ZFPOBI1-expressing mESCs. In control cells, the majority of RLTR10-associated TRIM28 peaks localize to RLTR10-int elements (92%), whereas only a small fraction overlap RLTR10 (5%) or RLTR10A (<1%) LTRs. Following ZFPOBI1 expression, TRIM28 occupancy is redistributed towards RLTR10 (18%) and RLTR10A (16%) LTRs, consistent with ZFPOBI1-dependent recruitment of TRIM28 to group a RLTR10 LTRs. **(I–J)** Heatmaps (top) and metaprofiles (bottom) showing HA, TRIM28 and H3K9me3 ChIP-seq signal across curated RLTR10E **(I)** and RLTR10F **(J)** elements aligned at the 5′ LTR boundary (±1 kb). Consistent with the low incidence of predicted ZFPOBI1 binding motifs within these ‘group b’ RLTR10 subfamilies, little to no HA-ZFPOBI1 enrichment is detected. TRIM28 occupancy and H3K9me3 levels are largely comparable between 3xHA-ZFPOBI1-expressing and empty-vector control mESCs, indicating an absence of ZFPOBI1-dependent recruitment at these loci.
